# Multi-parameter optimization of a novel anti-TROP2 MMAE antibody-drug-conjugate for solid tumor therapy

**DOI:** 10.3389/fonc.2026.1915652

**Published:** 2026-09-30

**Authors:** Laurence Fayadat-Dilman, Andrew B. Waight, Lauren Adams, Peyman Akbari, Minilik Angagaw, Jeanne Baker, Michelle Castor, Andy Chen, Daoyang Chen, Ming-Tang Chen, Ellen Chien, Jill E. Chrencik, Darcey Clark, James Dutko, Mehabaw Derebe, Alan Goggins, Thomas Griffiths, Mee Ra Heon, Marlene C. Hinton, SuChun Hsieh, Amy Hsu, Zhong L. Hua, Mark Ibrahim, Mengxuan Jia, Rebecca E. Johnson, Simon B. Lang, Su-Yang Liu, Daniel Malashock, Aleksandra Olow, Michael Oropallo, Andrea T. Partridge, Vin Penaflor, Elnaz Rasti Boroujeni, Zhao Ren, Karthik Sathiyamoorthy, Makiko Uchida, Derek Wiswell, Jane Xia, Peng Yang, Jennifer Yearley, Sultan Yilmaz, Yao Yu, John A. Flygare, Bernhard H. Geierstanger, Jianzhong Wen, W. Michael Seganish, Edward P. Bowman

**Affiliations:** 1 Discovery Biologics, Merck & Co., Inc., South San Francisco, CA, United States; 2 Pharmacokinetics, Dynamics, Metabolism and Bioanalytics (PDMB), Merck & Co., Inc., South San Francisco, CA, United States; 3 Quantitative Bioscience, Merck & Co., Inc., South San Francisco, CA, United States; 4 Quantitative Bioscience, Merck & Co., Inc., Boston, MA, United States; 5 Discovery Biologics, Merck & Co., Inc., Boston, MA, United States; 6 Discovery Chemistry, Merck & Co., Inc., South San Francisco, CA, United States; 7 Non-clinical Drug Safety, Merck & Co., Inc., South San Francisco, CA, United States; 8 Discovery Chemistry, Merck & Co., Inc., West Point, PA, United States; 9 Non-clinical Drug Safety, Merck & Co., Inc., West Point, PA, United States; 10 Discovery Oncology, Merck & Co., Inc., South San Francisco, CA, United States; 11 Data AI and Genome Science, Merck & Co., Inc., South San Francisco, CA, United States

**Keywords:** antibody-drug-conjugate, avidity tuning, hydrophobicity, MMAE, monoclonal antibody, solid tumors, stable linker, Trop2

## Abstract

TROP2 is expressed in many epithelial tumors including breast and lung cancers. It is a clinically validated ADC target based on approvals of sacituzumab govitecan for triple-negative breast cancer and datopotamab deruxtecan for HR-positive, HER2-negative breast cancer. An alternative cytotoxic approach was previously tested with PF-06664178/RN927C, an anti-TROP2 ADC with an auristatin payload, which entered clinical studies but could not be dosed efficaciously due to dose-limiting toxicities including skin rash, mucosal inflammation, and neutropenia. TroptimAb-SL-MMAE was engineered using an optimized anti-TROP2 sacituzumab antibody sequence (TroptimAb) to address issues related to hydrophobicity, non-specific uptake, and ADC developability. Sacituzumab was re_-_humanized and modified to enhance FcRn-mediated salvage which extended systemic half-life and potentially improving tumor distribution. Its affinity was avidity-tuned to preferentially bind cells with higher TROP2 expression to reduce on-target/off-tumor toxicity. An engineered cysteine in the Fc domain enabled generation of a homogeneous DAR2 ADC and shielded the linker-payload to reduce hydrophobicity and improve biophysical properties. Linker conjugation chemistry was also optimized to improve stability versus earlier cysteine-conjugated ADCs. We describe the preclinical profile of TroptimAb-SL-MMAE including its design, mechanism of action, anti-tumor activity, and pharmacokinetics.

## Introduction

Systemic chemotherapy utilizes a portfolio of cytotoxic small molecules such as microtubule inhibitors, topoisomerase inhibitors, DNA alkylating agents, and anti-metabolites which have a narrow therapeutic index. Common side effects include alopecia, reduced white blood cells, and gastrointestinal issues due to the cytotoxin activity on rapidly dividing cells in those tissues. Chemotherapy-induced peripheral neuropathy is also commonly seen with microtubule inhibitors and platinum-based DNA damaging agents. Targeted anti-cancer modalities aim to increase the therapeutic index by selectively targeting mutated proteins found only in the tumor cells (*e.g.* BRAF V600E kinase inhibitors), enhancing the immune system’s response to tumor neoantigens (e.g. anti-PD-1 antagonist pembrolizumab), or directing cytolytic T-cells to a single tumor antigen (e.g. DLL3xCD3 tarlatamab). Similarly, antibody-drug conjugates (ADC) seek to selectively enhance cytotoxic small molecule delivery to tumor-associated antigen positive (TAA+) tumor cells. There are currently fifteen approved ADCs that deliver either microtubule inhibitors, topoisomerase 1 (topo1) inhibitors, or DNA alkylators as cytotoxic payloads.

It is a misconception that ADCs follow Paul Ehrlich’s magic bullet hypothesis by selectively delivering cytotoxic payloads only to tumor cells while sparing normal tissues. Conventional antibody tissue distribution and catabolism principles also apply to ADCs because the modality is primarily comprised of IgG molecules (1). Moreover, ADC toxicities are similar to systemic chemotherapy suggesting that ADC toxicity is primarily driven by systemic exposure of the released cytotoxic payload. The benefit of ADCs is not that they are safer, but that greater efficacy can be achieved at tolerated doses compared to the efficacy that untargeted chemotherapy can achieve at their tolerated doses. It is notable that ADCs that bind different TAA but carry the same payload exhibit similar maximum tolerated doses (MTD) and similar toxicities (*i.e.* platform toxicity), supporting the above statement regarding off-target toxicities of ADCs. Additional on-target/off-tumor toxicities can arise via antigen-dependent targeting of the toxin to antigen-expressing normal tissue (2, 3).

The payload, the linker attaching the payload to the antibody, the attachment chemistry of the linker to the antibody, and the site in the antibody sequence where the linker is attached can impact the ADC’s platform toxicity profile. Linkers are categorized as either being cleavable or non-cleavable. Payloads are released from cleavable linkers by one or more mechanisms including protease activity, glyco-hydrolase activity, pH-dependent release, or disulfide reduction. Conversely, non-cleavable linkers require the full catabolism of the antibody to release the active payload. Linker-payload instability falls into two categories: antibody-linker instability or linker-payload instability. Antibody-linker instability involves deconjugation of the linker from the antibody leading to the loss of drug load from the ADC. Many ADCs use cysteine-maleimide chemistry to attach to native cysteines (interchain cysteine conjugation) on the antibody, and this is an area of known instability following dosing where significant amounts of linker-payload deconjugation can occur. Linker-payload instability refers to the premature release of payload due to inherent linker instability (e.g. pH-sensitive linkers) leading to systemic exposure of the untargeted payload (2, 3).

Antibodies and ADCs have two fundamental clearance mechanisms. Generic clearance is a mixture of macropinocytosis, Fcγ receptor (FcγR)-mediated uptake, and lectin receptor-mediated uptake. Target-mediated drug disposition (TMDD) is target specific and proportional to the level of the target’s surface expression. Only a small fraction of the ADC reaches the tumor to be catabolized to release payload (4, 5). The majority of ADC catabolism occurs via generic clearance mechanisms or through TMDD in normal tissues. ADC clearance via any mechanism leads to the generation of free payload and payload derivatives that redistribute throughout the body and are cleared via conventional small molecule elimination routes that can be payload specific (6–8).

TROP2, encoded by the TACSTD2 (tumor-associated calcium signal transducer 2) gene, is a transmembrane receptor within the EpCAM protein family. It is expressed in many epithelial tumors including breast and lung cancer and is not associated with hematologic cancers (9, 10). TROP2 obtained proof-of-concept as an ADC target with the approval of sacituzumab govitecan (for triple-negative breast cancer and hormone receptor (HR)-positive, human epidermal growth factor receptor 2 (HER2)-negative breast cancer (11). Datopotamab deruxtecan is the second TROP2 ADC approved for HR+/HER2- breast cancer (12).

Sacituzumab (also called hRS7) is the targeting antibody in sacituzumab govitecan and is linked to the cytotoxic topoisomerase 1 (topo1) inhibitor, SN-38. Sacituzumab govitecan binds to TROP2-expressing cells to releases SN-38 via the pH-dependent hydrolysis of the connecting maleimide-CL2A-carbonate linker. SN-38 non-covalently stabilizes the topo1:DNA complex and prevents the subsequent re-ligation of topo1-induced single strand DNA breaks. The resulting DNA damage leads to apoptosis induction and cell death. Many additional TROP2-topo1 ADCs are in clinical development including sacituzumab tirumotecan/MK-2870 that uses the same targeting antibody coupled via the sulfonyl-pyrimidine-CL2A-carbonate linker to release the topo1 inhibitor KL610023 (a belotecan derivative) in a pH-dependent manner (13). Similarly, ESG-401 uses the same antibody and SN-38 payload as sacituzumab govitecan but couples them using a protease-cleavable valine-citrulline dipeptide linker (14). A differentiated approach was taken for datopotamab deruxtecan (Dato-DXd) using a novel targeting TROP2 antibody linked to a more stable protease cleavable linker (tetrapeptide GGFG) to releases the topo1 inhibitor DXd (exatecan derivative) (15).

Targeting TROP2 with an orthogonal antimitotic mechanism has been attempted with PF-06664178/RN927C, an anti -TROP2 antibody conjugated with AcLys-valine-citrulline-Aur0101 (PF-06380101) linker-payload (16). The Aur0101 payload is in the auristatin class of potent tubulin polymerization inhibitors (17). Multiple PF-06664178 doses from 0.15 through 2.4 mg/kg were tolerated in the clinic; however, 3.6 mg/kg to 4.8 mg/kg doses were intolerable due to skin rash, mucosal inflammation, and neutropenia. Skin rash and mucosal inflammation observed in patients was also seen in non-human primate toxicology studies (16). Because TROP2 is highly expressed in the skin and esophagus, these are on-target toxicities and not due to off-target, PF-06380101 platform-related toxicities.

Monomethyl auristatin E (MMAE) is a commonly used antimitotic payload and the vedotin linker-MMAE platform has been clinically validated across multiple targets and indications. MMAE-ADCs primarily kill tumor cells by inhibiting tubulin polymerization leading to prolonged G2/M arrest; however, mitochondrial dysfunction is an important downstream contributor to cell death. Microtubule disruption can impair mitochondrial trafficking and bioenergetics, while prolonged mitotic stress activates the intrinsic apoptotic pathway through BAX/BAK-dependent mitochondrial outer membrane permeabilization, increased mitochondrial ROS, cytochrome c release, and caspase activation. MMAE exposure has also been associated with loss of mitochondrial membrane potential and increased mitochondrial ROS production. Thus, although microtubules are the direct target, efficient MMAE ADC cytotoxicity depends in part on activation of mitochondrial apoptotic machinery. Therapeutic MMAE ADC responses may also be modulated by mitochondrial quality control mechanisms including mitochondrial dynamics, mitophagy, and metabolic adaptation (18, 19).Common adverse reactions (occurring in >20-25% patients) seen with all approved vedotin ADCs are alopecia, hematologic toxicities, gastrointestinal toxicities, fatigue, and peripheral neuropathy (20, 21) (22, 23). These adverse reactions are independent of the ADC target because the toxicities are seen across both hematologic and solid tumor antigens (*i.e.* off-target vedotin platform toxicity). On-target/off-tumor toxicities occur in healthy organs expressing the targeted antigen. For example, Nectin-4 is expressed in the skin and eye (24) and enfortumab vedotin (Nectin-4-MMAE) causes skin rash and ocular disorders (21) (22, 25).

Our objective in the design of a novel TROP2 ADC is twofold. First, we aim to leverage the clinically validated MMAE payload which provides a complementary mechanism of action to the current clinical stage TROP2-topo1 but deploy a more stable linker that avoids MMAE toxicities due to DAR loss of the vedotin platform. Secondly, we sought to engineer the anti-TROP2 sacituzumab antibody sequence to address known or hypothesized tolerability issues with currently approved ADCs informed by PF-06664178 toxicity profile (16, 26). Sacituzumab was re-humanized to reduce its intrinsic elevated hydrophobicity profile, which could minimize non-specific uptake mechanisms following dosing and optimize the overall ADC developability properties. The antibody sequence was also avidity tuned to preferentially bind tumor cells that express higher TROP2 levels versus cells with lower TROP2 levels. The aim of the avidity tuning is to reduce on-target toxicity seen in healthy tissue with PF-06664178 (26). A cysteine residue was incorporated at S375 within the Fc CH3 domain to enable site-specific conjugation, and the surrounding Fc domain was utilized as a steric barrier to reduce premature linker deconjugation and proteolytic linker cleavage. Additionally, the conjugation site within the Fc domain shields the hydrophobic payload reducing the TroptimAb-SL-MMAE overall hydrophobicity (27). Overall, the rationally designed ADC demonstrates superior plasma stability and PK while releasing payload efficiently in mouse tumor xenograft models. This report describes the preclinical profiles of TroptimAb-SL-MMAE including its rational design, mechanisms of action, *in vitro/in vivo* anti-tumor activity, and pharmacokinetics.

## Materials and methods

### Analysis of TROP2 gene expression from the GTEx and TCGA databases using the Xena portal

A combined cohort of GTEx and TCGA RNA expression data was obtained from the University of California Santa Cruz Xena portal (accessed via https://xenabrowser.net; Goldman et al, 2020). The expression data was analyzed for TROP2 expression by the TACSTD2 gene using RSEM as TPM and UCSC Toil RNA-seq Recompute, with n=19,131. The GTEx database (accessed via http://www.gtexportal.org) is a publicly available deep sequencing (RNA-seq) data resource derived from normal human tissues. The GTEx data is generated from 17382 samples involving 948 individuals and 54 tissues. The TCGA database (accessed via the TCGA Research Network: https://www.cancer.gov/tcga) is a publicly available data resource which contains >20,000 primary cancer and matched normal samples spanning 33 cancer types that have been molecularly characterized. Data analysis and visualization were performed with R statistical programming language (4.1.0) with tidyverse (1.3.1) and ggplot2 (3.3.5) libraries. The samples were grouped by primary tissue or disease annotation provided by GTEx and TCGA. The limit of detection was set as 0.5 TPM gene expression, and the 10 TPM threshold was set as indicating medium/high expression level. These cutoff values are based on the European Bioinformatics Institute recommendations (https://www.ebi.ac.uk/gxa/FAQ.html).

### Analysis of TROP2 protein detection by immunohistochemistry

FFPE human and rhesus monkey normal and human tumor tissues from the Research Laboratories of Merck & Co., Inc., Rahway, NJ, USA internal tissue bank were used to determine TROP2 protein expression by IHC.

Tissues were stored as paraffin blocks or pre-cut sections at room temperature. A pathologist reviewed all samples for quality and histologic suitability. FFPE tissues were sectioned at 5 μm, baked at 60 °C for 45 min, and deparaffinized using the Stainer XL system (Leica Biosystems). Antigen retrieval was performed at 120 °C for 4 min. Endogenous peroxidase was quenched with 3% hydrogen peroxide for 10 min. Staining was carried out using the Autostainer 480S (Thermo Scientific, Michigan, USA). Slides were incubated with mouse anti-human TROP2 antibody (clone 01) or isotype control (0.3 µg/mL, 60 min), followed by HRP-conjugated secondary antibody (30 min).

Signal detection was performed using DAB chromogen, followed by hematoxylin counterstaining and mounting. TROP2 expression was quantified using a membrane H-score based on staining intensity (0–3+) and percentage of positive cells: H-score = (%1+) + 2×(%2+) + 3×(%3+).

### Re-humanization of sacituzumab

Humanization was performed using the Predict Humanizing Mutations function from the BIOIVA Discovery Studio (2021) software package. The native Kabat CDR regions along with the vernier residues were retained and grafted into subsequent human derived germline sequences. The Predict Humanizing Mutations protocol creates multiple acceptor sequences. One variant contains mutations suggested by the overall human antibody frequency statistics and is designated “frequent residue substitutions” (FRS). A second sequence variant contains mutations based on comparison to the closest matching germline gene sequence and is designated “germline substitutions” (GS). We also generated a third sequence that contains mutations produced by screening the mutation types found in the germline and, for those with frequency above 5%, for structural stability. Mutation energy calculations are performed using the CHARMM software (Harvard University, Cambridge, Massachusetts, USA) and molecular force field. Structures predicted to have stabilizing mutations by favorable energetic calculations are designated “best single mutations” (BSM).

### Hydrophobic interaction chromatography ProPac method

Antibody hydrophobicity was assessed using hydrophobic interaction chromatography (HIC). Samples (50 µg at 1 mg/mL) were mixed 1:1 (v/v) with 2 M ammonium sulfate in 100 mM sodium phosphate (pH 7.0) and incubated at ambient temperature for 20–30 min prior to injection.

Chromatography was performed on a ProPac HIC-10 column equilibrated in 1 M ammonium sulfate, 100 mM sodium phosphate (pH 7.0). Samples were eluted using an inverted gradient to 100 mM sodium phosphate (pH 7.0) at 30 °C over 45 min, with detection at 280 nm.

Retention times were analyzed using chromatography software. Relative hydrophobicity was determined by comparison to reference samples, with longer retention times indicating increased hydrophobicity.

### Determination of purity using reverse phase high-performance liquid chromatography

To determine purity using RP HPLC, 10 µg of purified sample was loaded onto a POROS R2/10 2.1 x 30 mm column. Mobile phase A is 0.2% trifluoroacetic acid in water and mobile phase B is 90% acetonitrile, 0.2% trifluoroacetic acid. The column is equilibrated with 30% of mobile phase B at 70 °C at a flowrate of 2 mL/minute. Samples were eluted with a linear gradient from 30% to 58% mobile phase B over 5 minutes. Chromatograms were collected at both 215 nm and 280 nm wavelengths, and integration of the absorbance at OD 280 nm trace was performed using ChemStation Rev. B.04.01 (Agilent Technologies, Inc., California, USA).

### Saturation mutagenesis yeast display library generation

Four site-saturation mutagenesis libraries were designed based on Chothia CDR definitions, targeting 31 heavy-chain CDR, 25 heavy-chain framework, 27 light-chain CDR, and 16 light-chain framework residues. Selected positions were randomized to all amino acids except cysteine, methionine, and tryptophan (except where present in the parental sequence).

Library fragments were synthesized with flanking homology regions for recombination into Fab yeast display vectors. Each library (50 ng) was PCR-amplified and purified by ultrafiltration. Heavy- and light-chain vectors were restriction digested (NheI/XhoI or NheI/BsiWI) and gel purified prior to transformation.

Libraries were introduced into *Saccharomyces cerevisiae* strains (yBDB360 for light chain and yBDB499 for heavy chain) by electroporation. Cells were grown to OD600 ~1.6, washed, chemically treated, and resuspended in electroporation buffer. For each transformation, 80 OD600 cell equivalents were co-electroporated with 4 µg linearized vector and 12 µg library DNA. After 1 h recovery, cells were plated on selective media to determine library size (>5 × 10^7^ transformants). Parent heavy and light chains were transformed in parallel.

For diploid display library generation, library strains were mated with strains expressing the corresponding parental chain. Equal cell densities (150 OD600) were combined, plated on YPD agar, and incubated for 6 h at 30 °C. Cells were then selected in double-selection media. Mating efficiencies exceeded 1 × 10^8^ diploids. A parental control clone was generated by mating parental heavy and light chain strains.

### Yeast display selection for sacituzumab reduced affinity variants to TROP2

Yeast display libraries and the parent clone were cultured in minimal dextrose selective media at 30 °C for 24 h, followed by growth overnight in minimal raffinose media at 0.5 OD600. Fab display was induced by seeding at 1 OD600 in galactose/raffinose selective media supplemented with 1.8 µg/mL PMTi4 and incubating at 24 °C for 24 h.

Approximately 5 × 10^7^ cells from each library and the parent clone were washed and incubated with biotinylated human TROP2 (100, 30, or 10 nM) for 1 h at 30 °C. After washing, cells were labeled with anti-human kappa Alexa Fluor 647, Neutravidin-PE, and YOYO-1 iodide for 20 min on ice, protected from light. Cells were washed and analyzed by fluorescence-activated cell sorting (FACS). Library populations were compared with the parent clone, and sorting gates were applied across all antigen concentrations to isolate variants with reduced binding. Sorted cells were plated on selective media and grown for 3 days.

Individual colonies were screened by colony PCR using heavy- and light-chain-specific primers. Amplified products were sequenced, and variants were analyzed to identify enriched mutations for subsequent production and characterization.

### SPR for assessment of binding affinity of anti-TROP2 variants to TROP2 (display)

Anti-TROP2 antibodies were captured by an anti-human Fc antibody immobilized on a CM4 biosensor chip. Binding assays were performed using HBS-EP+ buffer containing 10 mM HEPES, 150 mM NaCl, 3 mM EDTA, 0.05% surfactant P20, and 2% DMSO, pH 7.6. Human TROP2 was injected at increasing 3-fold concentrations (0, 0.01, 0.04, 0.11, 0.33, and 1 µM) and allowed to sequentially bind to the immobilized ligand for 420 seconds at a flow rate of 30 µL/min at 37 °C. After association of the highest ligand concentration, an 1800 second dissociation period followed. The assay was performed on a Biacore™ T200 instrument (Cytiva, Marlborough, Massachusetts, USA). The data were fitted to a 1:1 binding model using Cytiva’s Insight Evaluation software. In this format, the apparent affinity (KD) reported as the interaction of antigen to captured antibody may be bivalent.

### Quantitation of cell surface TROP2 by flow cytometry

Cells were washed once with PBS (without calcium or magnesium) and then treated with Enzyme-Free Cell Dissociation Buffer at 37°C until detached. Following dissociation, the cells were washed in assay buffer (PBS with 2% FBS, 0.04% sodium azide), filtered through a 40 µm cell strainer, counted, and prepared for 2E5 viable cells in 100 µL of assay buffer per tube or well. PE-conjugated anti-human TROP2 antibody at 40 µg/mL was added to the cells and incubated on ice for 30 minutes. The cells were then washed and resuspended in assay buffer with 1 µg/mL DAPI for labelling of dead cells. Quantitation of TROP2 copy number was performed on the live cell population. BD Quantibrite™ Beads were prepared following the manufacturer’s protocol and used as a calibrator. Data were acquired on the Discovery Fortessa™ (Becton Dickinson, San Jose, California, USA) flow cytometer and analyzed with FlowJo™ software (Becton Dickinson) for an estimation of antibodies bound per cell.

### Cell-based ELISA Using BxPC-3, MDA-MD-231-KWL, and EMT6 cells

Sacituzumab variants’ binding potency was evaluated on TROP2-high BxPC-3 (~456K TROP2 molecules per cell), TROP2-low MDA-MB-231 (~43K TROP2 molecules on the surface), and TROP2-negative mouse EMT6 cells in a cell-based ELISA assay. Cells were plated in duplicate onto 96-well plates and antibodies diluted 5-fold from a starting concentration of 30 µg/mL (200 nM) in cell culture medium were added and allowed to incubate for one hour. After 3 washes, a labeled secondary anti-human IgG was added for 1 hour at 1 µg/ml, washed 3 times, and absorbance was measured at 450 nm. The EC50 was measured along with the AUC for each cell line. The differential between the AUC of BxPC-3 and MDA MB-231 binding (ΔAUC/AUC (BxPC-3)) was used to identify mutants which bind with higher avidity TROP2-high versus TROP2-high cells *in vitro*.

### Assessment of aggregation using ultra-high-performance size-exclusion chromatography

To determine aggregation by UPSEC, 5 µg of purified sample was injected onto a BEH200 (Waters Acquity BEH200 SEC, 1.7 μm, 4.6 x 150 mm) size exclusion column that was equilibrated with 100 mM sodium phosphate, 200 mM sodium chloride pH 7.2, at 0.5 mL/minute using a Waters H-Class UPLC (Waters Corporation, Milford, Massachusetts, USA). Chromatograms were collected at both 215 nm and 280 nm wavelengths, and integration of the absorption at OD 280 nm (wavelength) trace was performed using Empower 3 (Waters Corporation).

### Determination of purity by sodium dodecyl sulfate capillary electrophoresis

Protein Express Sample Buffer, PerkinElmer) containing either iodoacetamide (non-reduced) or dithiothreitol (reduced). Samples were incubated at 70 °C for 20 min, diluted with water, and analyzed on a LabChip GXII system (PerkinElmer) using an HT Protein Express Chip. Electropherograms were collected by fluorescence detection and analyzed using LabChip GX software to determine purity profiles.

### Assessment of melting point and aggregation temperature using nano differential scanning fluorimetry

Thermal stability was assessed by nano differential scanning fluorimetry (NanoDSF) using intrinsic tryptophan and tyrosine fluorescence). Samples (~10 µL at 1 mg/mL) were loaded into capillaries and subjected to a temperature ramp from 20 °C to 95 °C at 1 °C/min using a Prometheus NT.48 instrument (Nanotemper, Germany).

Protein unfolding was monitored by fluorescence emission at 330 nm and 350 nm, and unfolding curves were generated from the F350/F330 ratio. The melting temperature (Tm) was defined as the midpoint of thermal unfolding and determined from the inflection point of the curve.

Aggregation temperature (Tagg) was determined by monitoring light scattering, with increased scattering indicating aggregate formation. Tagg corresponded to the onset of aggregation.

### Analysis using hydrophobic interaction chromatography

Hydrophobicity of ADCs and unconjugated antibodies was assessed by hydrophobic interaction chromatography using a TSKgel Butyl-NPR column. Samples (50 µg at 1 mg/mL) were mixed 1:1 (v/v) with 1.5 M ammonium sulfate in 50 mM sodium phosphate (pH 7.0) and filtered (0.22 µm) prior to analysis.

Samples (25 µg ADC or 10 µg antibody) were injected onto a column equilibrated in 1.5 M ammonium sulfate, 50 mM sodium phosphate (pH 7.0) and eluted using an inverted gradient to buffer without ammonium sulfate. Chromatography was performed at ambient temperature over ~50 min with detection at 280 nm.

Retention times were determined from chromatograms and compared to reference samples. Hydrophobicity was inferred from retention time, with longer elution corresponding to increased hydrophobicity. Retention time shifts between ADC and unconjugated antibody were also calculated.

### BxPC3 efficacy model-avidity tuned mutant screening

Procedures involving the care and use of animals in the study were reviewed and approved by the Institutional Animal Care and Use Committee at the Research Laboratories of Merck & Co., Inc., Rahway, NJ, USA (a division of MSD). During the study, the care and use of animals were conducted in accordance with the principles outlined in the guidance of the Association for Assessment and Accreditation of Laboratory Animal Care (AAALAC), the Animal Welfare Act, the American Veterinary Medical Association (AVMA) Euthanasia Panel on Euthanasia, and the Institute for Laboratory Animal Research (ILAR) Guide to the Care and Use of Laboratory Animals.

Tumor volumes were measured twice weekly using calipers and calculated as TV = a × b²/2, where *a* and *b* represent long and short tumor diameters. Tumor growth inhibition (TGI) was calculated as TGI (%) = (1 − T/C) × 100. Animals exceeding 2000 mm³ tumor volume were euthanized. Body weight was monitored twice weekly and expressed relative to baseline. BxPC-3 human pancreatic tumor cells were cultured in RPMI 1640 with 10% FBS and maintained under standard conditions. Cells in exponential growth were harvested and inoculated subcutaneously (1 × 10^7^ cells, 0.2 mL in PBS/Matrigel) into BALB/c nude mice. Treatment was initiated when tumors reached ~190–195 mm³. Efficacy cohorts included 10 mice per group, and pharmacokinetic cohorts included 3 mice per group.

### Mouse and NHP PK sample collection and analysis

All animal procedures were conducted according to the highest ethical standards using procedures approved by the Merck & Co., Inc., Rahway, NJ, USA IACUC. For mouse PK analysis, blood samples were collected via tail vein at 2 h and days 1, 3, 7, 10, and 14 into EDTA tubes (Becton Dickinson), centrifuged (2500 × g, 4 °C, 6 min), and plasma stored at −80 °C. Tumors were collected 3 days post-dose, homogenized at 4 °C (Precellys, Bertin), and analyzed for payload levels. For non-human primates, sacituzumab variants were administered intravenously to rhesus monkeys (n = 2). Blood was collected into serum separator tubes, allowed to clot, centrifuged, and serum stored at −80 °C.

FcRn transgenic (Tg32) mice were used to evaluate YTE-mediated effects. Blood and tissues were collected 14 days post-dose for measurement of conjugated antibody (cAb) and tissue MMAE.

cAb and total antibody (tAb) concentrations were quantified using electrochemiluminescence immunoassays. For cAb, plates were coated with anti-MMAE antibody, blocked, and incubated with diluted samples and standards. Bound antibody was detected using SULFO-TAG™ anti-human IgG and read on a SECTOR Imager (Meso Scale Discovery). The tAb assay used a similar format with anti-human Fc capture. Concentrations were calculated from standard curves.

MMAE levels in plasma and tumor homogenates were quantified by LC-MS/MS following protein precipitation with acetonitrile and centrifugation. Pharmacokinetic parameters were determined by non-compartmental analysis, and concentration–time profiles were generated using standard software.

### Generation of mouse EMT6 cells engineered to express different TROP2 receptor densities

The human TROP2 gene (NM_002353.3) was synthesized by Twist Bioscience and subcloned by Alstem (Richmond, CA) into the EcoR1 and BamH1 restriction sites of pLenti-EF1a-MCS vector (no selectable marker) to create the lentiviral expression plasmid pLenti-EF1a-hTROP2. HEK293FT cells (5 x 10^6) were seeded in a 10 cm dish and transfected with the pLenti-EF1-hTROP2 plasmid to package lentivirus particles. The supernatant containing virus was harvested two days following transfection and concentrated viral particles were stored at -80 °C. Mouse EMT6 cells were cultured in alpha MEM with 10% FBS and penicillin/streptomycin. EMT6 cells were plated in 12-well plate at 1 x 10^5 cells per well and transduced with pLenti-EF1a-hTROP2 lentivirus at MOI of 1 or 10 the following day. The cell culture medium was replaced daily during cell expansion. Cell pools were stained with PE-Mouse Anti-Human TROP2 antibody (BD Pharmingen™ Cat # 564837) to confirm TROP2 expression. Subsequently, the stable cell pools were seeded in 96-well plates to a final concentration of 0.5 cells per 100 μl per well by limiting dilution. Single cell colonies were monitored, expanded, and TROP2 expression evaluated by flow cytometry. TROP2 receptor density determined in selected EMT6.hTROP2 single clones as outlined above. The EMT6.hTROP2^184K^ and EMT6.hTROP2^37K^ was selected for further analysis.

### Use of EMT6 transfectants with varying levels of human TROP2 to evaluate how the *in vivo* potency of sacituzumab and sacituzumab(Y53D) is altered by TROP2 surface density

EMT6.hTROP2^184K^ and EMT6.hTROP2^37K^ murine mammary tumor cells were engineered to have human TROP2 at 1.84 × 10^5^ or 3.7 x 10^4^ surface copies per cell. Cells were maintained *in vitro* as suspension in DMEM medium supplemented with 10% FBS in a humidified incubator at 37 °C, 5% CO2. Tumor cells in an exponential growth phase were harvested and counted for tumor inoculation. Each animal was inoculated SC into the left flank with a single cell suspension of 5 × 10^6^ tumor cells per 0.1 mL in 1:1 PBS with Matrigel. Animals were weighed and assigned to treatment groups using a randomization procedure. Group assignment and treatment were started at Day 0 when the mean tumor volume reached approximately 100 mm3. Dosing volume was 5 mL/kg adjusted based on body weight; for example, a 20 g mouse would receive 0.1 mL.

### Expression and Purification of TroptimAb and generation of TroptimAb-SL-MMAE

At the screening stage, sacituzumab and avidity-tuned variants were expressed in HEK293 cells and purified by Protein A chromatography. For ADC generation and developability studies, antibodies were expressed in stable CHO GS cells and purified using a three-step process. First, samples were captured on Protein A resin. Second, flow-through from anion exchange chromatography was subjected to on-resin reduction with DTT followed by re-oxidation with DHAA, and eluted under acidic conditions. Third, polishing was performed by cation exchange chromatography to remove aggregates and impurities, with purity assessed by UP-SEC.

Purified antibodies were dialyzed into formulation buffer (10 mM histidine, 9% sucrose, pH 6.5). Site-specific conjugation to generate TroptimAb-SL-MMAE was achieved using cysteine–maleimide chemistry at the engineered S375C Fc position.

### Determination of DAR of TroptimAb-SL-MMAE by mass spectrometry

Drug-to-antibody ratio (DAR) was determined by LC-MS using a time-of-flight instrument. TroptimAb-SL-MMAE was diluted to 0.2 µg/µL in 50 mM ammonium bicarbonate. For reduced analysis, samples were treated with TCEP and incubated at 37 °C for 30 min.

For intact and reduced mass analysis, 5 µL of sample was injected onto a PLRP-S column (1000 Å, 5 µm, 50 × 2.1 mm) at 80 °C and 0.5 mL/min. Samples were separated using linear gradients of acetonitrile/formic acid (20–75% or 15–75% over 11–13 min).

Mass spectra were acquired over m/z ranges of 800–7000 (intact) or 500–3200 (reduced) and deconvoluted using standard software. DAR values were calculated from deconvoluted spectra.

### Assessment of TroptimAb-SL-MMAE binding kinetics to human TROP2 by surface plasmon resonance

Binding affinity of TroptimAb-SL-MMAE to human, rhesus/cynomologus monkey, canine, rat, and mouse TROP2 were determined by SPR using a Biacore T200 biosensor (Cytiva, MA). An anti-human IgG Fc antibody was immobilized on a CM5 chip by amine coupling. TroptimAb-SL-MMAE (10 nM) was captured, and assays were performed at 25 °C in HBS-EP+ buffer.

Human TROP2 was injected as a 5-point, 3-fold dilution series starting at 300 nM. Association and dissociation phases were monitored for 180 s and 600 s, respectively. Surfaces were regenerated with glycine (pH 1.5) between injections.

Data were double referenced and analyzed using Biacore Insight software. Association (ka) and dissociation (kd) rate constants were determined and used to calculate the equilibrium dissociation constant (KD = kd/ka) using a 1:1 binding model. Reported affinities are apparent values reflecting potential bivalent interactions.

Binding affinity of TroptimAb to human TROP2 was determined by SPR using a Biacore T200 biosensor (Cytiva, MA). Trop-2 was directly immobilized on a CM5 chip by amine coupling at pH 5.5 to achieve a low level, ~13RU, to mimic non-cancer cells. TroptimAb and sacituzumab antibodies were injected 5 concentrations, 3-fold dilution series at the top concentration of 100nM, while TroptimAb-Fab and sacituzumab Fab were injected at the top concentration of 500nM. Immobilization and kinetic assays were performed at were performed at 25 °C in HBS-EP+ buffer.

Data was double referenced and analyzed using Biacore Insight software. Association (ka) and dissociation (kd) rate constants were determined and used to calculate the equilibrium dissociation constant (KD = kd/ka) using a 1:1 binding model. Reported affinities are apparent values reflecting potential bivalent interactions.

### *In silico* immunogenicity risk assessment

*In silico* immunogenicity analysis of amino acid sequences of antibody constructs was performed using EpiMatrix v1.2 with DR9 software (EpiVax, Inc, Providence, Rhode Island, USA). The software employed algorithms to identify epitopes which are putative 9-mer MHC binding sequences and to generate HLA-binding predictions to nine commonly occurring HLA-DRB1 haplotypes. The primary amino acid sequence of the antibody construct was broken down into 9-mer peptide frames and assessed for the probability of binding to the various HLA alleles. Epitopes which bind at least half of the HLA supertype alleles identified via EpiMatrix were used to predict HLA binding at the population level. An estimate of the immunogenic potential for a given protein was provided with a score based on normalization of the number of predicted epitopes to sequence length using the Protein Immunogenicity Assessment Tool in the software.

### Expression, purification, crystallization of the human TROP2 ectodomain complexed with TroptimAb’s Fab, and crystallography analysis

The extracellular domain of Trop2 (residues 31–274) was expressed in Sf21 insect cells and purified as previously described (PDB ID: 7PEE). Briefly, the TROP2 ectodomain was harvested from the culture supernatant and purified by immobilized metal affinity chromatography (IMAC). The affinity tag was removed by TEV protease cleavage followed by dialysis and a reverse IMAC step to remove the cleaved tag and protease. The purified TROP-2 ectodomain was then complexed with Fab fragments of TroptimAb or sacituzumab and subjected to size-exclusion chromatography (SEC) for final purification. The resulting complex was concentrated to 15 mg/mL, flash-frozen in liquid nitrogen, and stored until further use.

Crystallization was performed using the sitting-drop vapor diffusion method at 4 °C. Drops were prepared by mixing protein complex with a precipitant solution containing 20% (w/v) PEG 3350 and 0.2 M ammonium phosphate (NH4)2HPO4). Crystals appeared over the course of 7 days. X-ray diffraction data were collected at NSLS (FMX-2; 17-ID-2 beamline). Data processing was carried out using autoPROC for global scaling and integration. The structure was solved by molecular replacement with MolRep in the CCP4 suite, using the Trop2 ectodomain (PDB ID: 7PEE) and Fab structures as search models. The initial model was refined through iterative cycles of manual rebuilding in Coot27 and automated refinement with BUSTER28,29 resulting in the final structural model.

### TroptimAb-SL-MMAE *in vitro* plasma stability

Gender-pooled heparin plasma (mouse, rat, monkey, human; BioIVT) was incubated with TroptimAb-SL-MMAE (50 µg/mL) at 37 °C. Aliquots were collected at 0, 1, 3, 5, and 7 days and stored at −20 °C prior to analysis.

Released MMAE was quantified by LC-MS/MS following protein precipitation with acetonitrile (1:2 v/v) containing internal standard, vortexing, and centrifugation (4000 × g, 10 min). Percent MMAE release was calculated relative to total conjugated MMAE at time 0.

For DAR assessment, TroptimAb-SL-MMAE was enriched from plasma samples using immunoaffinity capture (AssayMAP, Agilent). Biotinylated TROP2 or anti-human IgG antibodies (Acro Biosystems; Southern Biotech) were immobilized on streptavidin resin tips for capture. Samples were diluted in TBS with Rapigest, washed, and eluted in formic acid/acetonitrile prior to LC-MS analysis.

DAR values were calculated from intact mass spectra and normalized to time 0 to determine percentage DAR remaining.

### *In vitro* TroptimAb-SL-MMAE cytotoxicity assessment during neutrophil differentiation

Human hematopoietic stem cells (CD34+) were obtained from Stem Cell Technology (cat# 70002) and expanded for three days in StemSpan SFEM II serum-free basal medium (StemCell Technologies, cat# 9605) supplemented with StemSpan CC100 cytokine cocktail (cat# 02690). Neutrophil differentiation was performed by incubating expanded HSCs for 4 days in StemSpan SFEM II serum free basal medium supplemented with 50 ng/ml SCF, 100 ng/mL Flt-3L, 5 ng/mL IL-3, 5 ng/mL GM-CSF, and 5 ng/mL G-CSF (R&D Systems, cat# 255-SC-010, 308-FK-025, 203-IL-010, 215-GM-010, 214-CS-025 respectively). Cells were then incubated for 4 days in StemSpan SFEM II serum free basal medium supplemented with 5 ng/mL IL-3 and 30 ng/mL G-CSF. Neutrophil differentiation was confirmed by assessing CD34, CD66b and CD15 expression by flow cytometry. Differentiated neutrophils were incubated in StemSpan SFEM II serum-free basal medium supplemented with 30 ng/mL G-CSF and increasing concentrations of MMAE payload or ADCs. Viability measured by ATP luciferase (Promega, cat# G9682) was determined after six days.

### *In vitro* TroptimAb-SL-MMAE cytotoxicity assessment during megakaryocyte differentiation

Human hematopoietic stem cells (CD34+) were obtained from Stem Cell Technology (cat# 70002) and expanded for three days in StemSpan SFEM II serum-free basal medium (StemCell Technologies, cat# 9605) supplemented with StemSpan CC100 cytokine cocktail (cat# 02690). Expanded HSCs were incubated with StemSpan SFEM II serum-free basal medium and supplemented with StemSpan Megakaryocyte Expansion Supplement (StemCell Technologies, cat# 02696) and ADCs or MMAE alone for 6 days. Differentiation of Megakaryocytes was confirmed by measuring the expression of CD34, CD42, and CD41 by flow cytometry and viability was determined by ATP luciferase.

### *In vitro* TroptimAb-SL-MMAE linker stability assessment using differentiated neutrophil conditioned media

Human hematopoietic stem cells (CD34+) were obtained from Stem Cell Technology (cat# 70002) and expanded for three days in StemSpan SFEM II serum-free basal medium (StemCell Technologies, cat# 9605) supplemented with StemSpan CC100 cytokine cocktail (cat# 02690). Neutrophil differentiation was performed by incubating expanded HSCs for 4 days in StemSpan SFEM II serum free basal medium supplemented with 50 ng/ml SCF, 100 ng/mL Flt-3L, 5 ng/mL IL-3, 5 ng/mL GM-CSF, and 5 ng/mL G-CSF (R&D Systems, cat# 255-SC-010, 308-FK-025, 203 IL 010, 215-GM-010, 214-CS-025 respectively). Cells were then incubated for 4 days in StemSpan SFEM II serum-free basal medium supplemented with 5 ng/mL IL-3 and 30 ng/mL G-CSF. Following this 4-day differentiation, the neutrophils were incubated in StemSpan SFEM II serum-free basal medium supplemented with 30 ng/mL G-CSF for 6 days before the cell culture was centrifuged at 300 x g for 5 min to collect cell-free conditioned media for use in the Linker Stability Assessment. ADCs and MMAE were incubated in this media for 24 hrs at 37oC before the media was collected and stored at -70oC for LC-MS analysis.

### TroptimAb-SL-MMAE *in vitro* cytotoxicity

The cytotoxicity of TroptimAb-SL-MMAE on TROP2-expressing cells with different TROP2 surface densities was assessed using the CellTiter-Glo^®^ 2.0 Assay. Isotype Mab-SL-MMAE (anti-RSV-MMAE) and MMAE were included as controls. Human cancer cell lines (AGS, NCI-N87, Calu-3, HCC78, HCC1806, JIMT-1, MCF-7, MDA MB-231, BxPC3, SW620) and normal primary cells (H-6046, PCS-200, PCS-300, PCS-301) were seeded in growth medium in white 384-well microplates at 1500 or 300 cells per well, respectively, on Day 0 and incubated overnight at 37 °C, 5% carbon dioxide. On Day 1, cells were treated with a serial dilution of TroptimAb-SL-MMAE, anti-RSV-SL-MMAE, or MMAE and incubated at 37 °C, 5% carbon dioxide for 4 days. On Day 5, CellTiter-Glo^®^ 2.0 Reagent was added to the cells under gentle shaking for 2 to 3 minutes at ambient temperature to induce cell lysis, followed by 5 additional minutes without shaking to stabilize the luminescent signal. Luminescence was detected on an EnVision^®^ microplate reader (PerkinElmer, Waltham, Massachusetts, USA). The cytotoxicity assay data were processed using TIBCO Spotfire (TIBCO Spotfire, Inc, Palo Alto, California, USA), which applies a 4-parameter logistic fit based on the Levenberg Marquardt algorithm model 205. The data are presented as the geometric means of the EC50 and may include data from different lots.

### TroptimAb-SL-MMAE full dose response in BxPC3 efficacy model and PK analysis

BxPC-3 cells were cultured in RPMI 1640 supplemented with 10% FBS at 37 °C and 5% CO_2_. Cells in exponential growth were harvested and inoculated subcutaneously (1 × 10^7^ cells in 0.2 mL PBS/Matrigel) into BALB/c nude mice.

Treatment was initiated when tumors reached ~190 mm³. Efficacy cohorts consisted of 7 groups (n = 10/group). Plasma PK time points were serially collected from five mice per time point from mice in the efficacy cohort. In addition, three extra mice were included in certain cohorts to collect day 3 terminal tumor tissues.

Tumor volume comparisons were analyzed by first assessing variance homogeneity (Bartlett’s test). Parametric (one-way ANOVA with Tukey’s and Dunnett’s *post hoc* tests) or non-parametric (Kruskal–Wallis with Dunn’s’s test) methods were applied accordingly.

TroptimAb-SL-MMAE PK analysis followed the same statistical approach.

### TroptimAb-SL-MMAE efficacy in PDX models

Patient-derived xenograft (PDX) model assessment was performed at Crown Biosciences; PDX models were chosen based on TROP2 gene expression. Fresh tumor tissues from mice bearing established primary tumors were harvested and cut into small pieces (approximately 2 to 3 mm in diameter) and used for inoculation. Tumor fragments from donor tumor bearing mice were inoculated SC in the right flank of corresponding BALB/c nude or NOD/SCID mice for tumor development. Group assignment and treatment were started at Day 0 when the mean tumor volume reached approximately 200 mm3. Dosing volume was 5 mL/kg adjusted based on body weight; for example, a 20 g mouse would receive 0.1 mL. All NOD/SCID mice received 10 mg/kg human IVIG at Day -1 to normalize serum Ig basal levels with those anticipated for BALB/c nude mice.

To compare tumor volumes of different groups at a pre-specified day, Bartlett’s test was used first to check the assumption of homogeneity of variance across all groups. When the p-value of Bartlett’s test was ≥0.05, one-way ANOVA was run to test overall equality of means across all groups. If the p-value of the one-way ANOVA was <0.05, *post hoc* testing was performed by running Tukey’s HSD (honest significant difference) tests for all pairwise comparisons, and Dunnett’s tests for comparing each treatment group with the vehicle group. When the p-value of Bartlett’s test was <0.05, Kruskal-Wallis test was run to test overall equality of medians among all groups. If the p-value of the Kruskal-Wallis test was <0.05, *post hoc* testing was performed by running Dunn’s’s non-parametric test for all pairwise comparisons or for comparing each treatment group with the vehicle group, both with single step p-value adjustment.

### Immunohistochemical analysis of PDX models

Tumors from three mice per model were collected at randomization and processed into FFPE blocks for TROP2 IHC analysis. Sections (4 μm) were baked at 60 °C, deparaffinized using the Stainer XL system (Leica Biosystems), and subjected to heat-induced antigen retrieval at 120 °C for 4 min.

Endogenous peroxidase was quenched with 3% hydrogen peroxide. Staining was performed using an Autostainer 480S (Thermo Scientific). Slides were incubated with rabbit anti-TROP2 antibody (clone EPR20043, 0.5 µg/mL, 60 min), followed by HRP-conjugated secondary antibody (30 min).

Signal was visualized with DAB, and slides were counterstained with hematoxylin, mounted, and evaluated by a pathologist.

TROP2 expression was quantified using a membrane H-score based on staining intensity (0–3+) and percentage of positive cells:

H-score = (%1+) + 2×(%2+) + 3×(%3+).

### TroptimAb-SL-MMAE + Anti-PD-1 combination benefit in PD-1 resistant syngeneic tumor model EMT6.hTROP2^184K^

The syngeneic mouse EMT6 murine mammary tumor model is resistant to PD-1 blockade mechanism and therefore is an optimal model to study the combination of novel agents with PD-1 blockade. EMT6 cells were engineered to have high expression of human TROP2 at 1.84 × 10^5^ surface copies per cell (EMT6.hTROP2^184K^, clone 62). Cells were maintained *in vitro* as suspension in DMEM medium supplemented with 10% FBS in a humidified incubator at 37 °C, 5% CO_2_. EMT6.hTROP2^184K^ tumor cells in an exponential growth phase were harvested and mice inoculated SC into the left flank with a single cell suspension of 5 × 10^6^ EMT6.hTROP2^184K^ tumor cells per 0.1 mL in 1:1 PBS with Matrigel. Animals were randomized into treatment groups of 10 mice each when the mean tumor size reached approximately 143 mm^3^. A sub-efficacious dose of TroptimAb-SL-MMAE was selected to allow for the evaluation of enhanced antitumor activity in the combination groups. The muDX400 dose selected, 5 mg/kg, is the maximum dose used in anti-PD-1 sensitive syngeneic tumor models. Approximately nine weeks post-tumor regression, all tumor-free mice were re-inoculated with 1 × 10^6^ EMT6.hTrop2^184K^ tumor cells into the left flank and an additional 10 naïve mice were challenged for the first time. The “re-challenge” mice were monitored for approximately an additional 6 weeks.

## Results

### Design of a stable MMAE linker payload with a protease cleavable linker

MMAE has been extensively studied as part of the vedotin platform used by several approved ADCs. Our interest in MMAE came from the understanding that the vast majority of TROP2 targeting ADCs leveraged topo1 inhibitors. We therefore prioritized a payload that was differentiated with regard to its mechanism of action (microtubule inhibitor) to allow payload cycling with TROP2-topo1 ADCs. This feature would enable patients who did not initially respond to or lost response to TROP2-topo1 ADC to receive a TROP2 antimitotic ADC and potentially overcome the previous lack of response that was due to intrinsic or acquired resistance to the topo1 payload. Well established cysteine-maleimide conjugation chemistry was used with the maleimide designed to enable facile succinimide hydrolysis after conjugation (**Figure 1A**). Rapid hydrolysis of the succinimide ring is a useful method to prevent antibody-linker deconjugation that plagued early ADC designs (28, 29). The linker-payload has a lysosomal protease cleavable linker (alanine-alanine) and a self-immolative para-amino benzyl carbamate (PABC) connection to the MMAE payload to achieve traceless MMAE release (*i.e.* maleimidolpropionyl-alanine-alanine-p-aminobenzyloxycarbonyl-MMAE; stabilized linker (SL-MMAE)). The chosen linker maintains stability in plasma but also ensures efficient payload release upon internalization and catabolism within TROP2 expressing tumor cells (*vida infra*).

**Figure 1:**
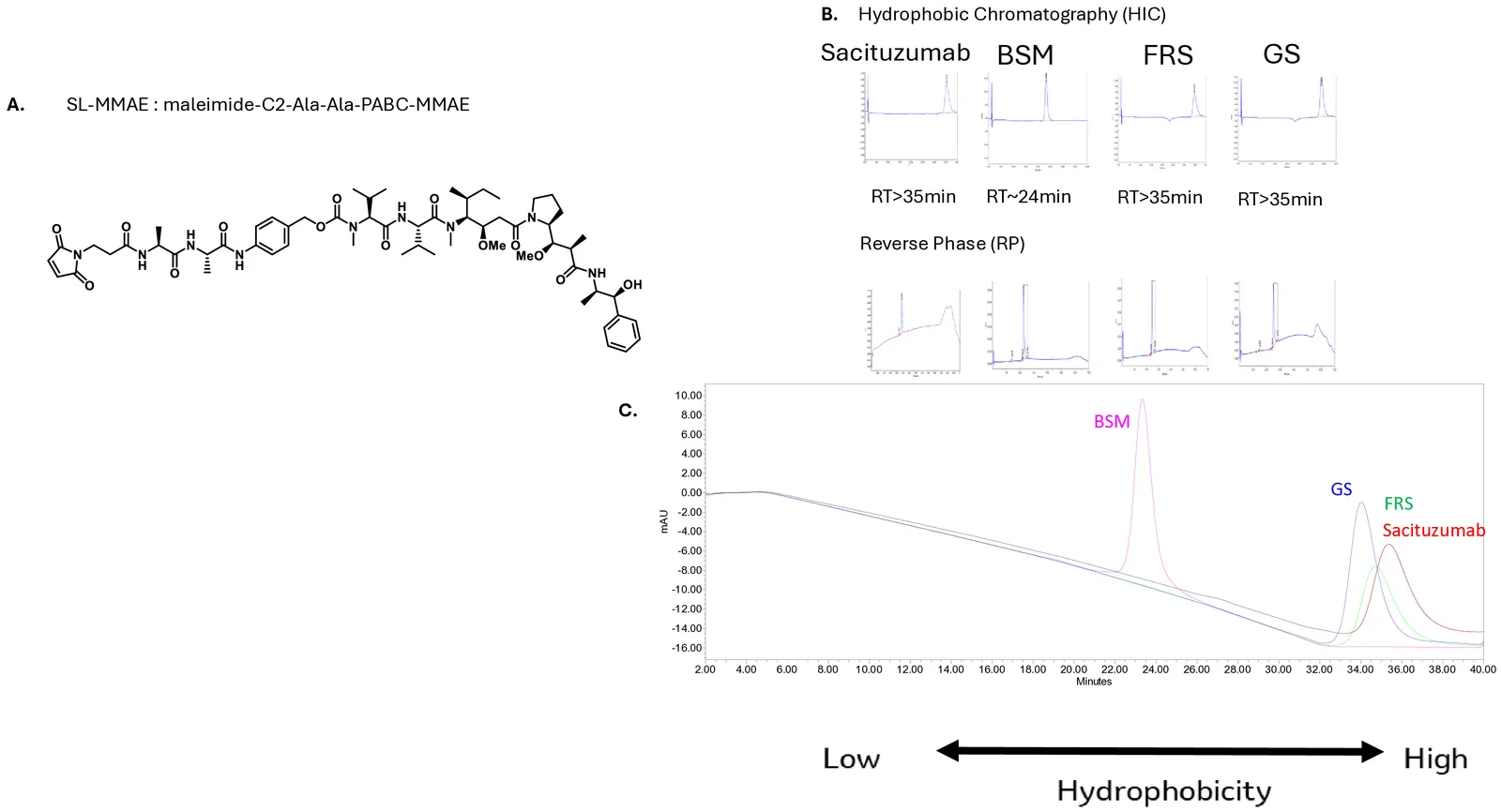
MMAE linker payload and sacituzumab re-humanization. **(A)** SL-MMAE (maleimidolpropionyl-alanine-alanine-p-aminobenzyloxycarbonyl-MMAE), **(B)** Elution profile of sacituzumab, BSM, FRS, and GS re-humanized variants by HIC using Propac method/column and RP chromatography run at 80 °C, **(C)** Overlay of HIC profiles for Sacituzumab, BSM, FRS, and GS variants.

### Principles to design an optimized TROP2 ADC

Several orthogonal protein engineering approaches were employed in order to optimize key properties of the TROP2 antibody sacituzumab and its ADC derivative to increase the tolerability of a TROP2-SL-MMAE ADC. First, sacituzumab was re-humanized to reduce its hydrophobicity profile to minimize non-specific ADC uptake into non-target expressing cells and to increase the humanness of the antibody. Second, decreasing binding to TROP2-low (<50K surface copy number) cells by decreasing sacituzumab’s affinity and selecting a variant that binds TROP2-high (>150K surface copy number) cells using primarily avid interactions. Third, select amongst the potential avidity tuned mutations for the variant with the lowest hydrophobicity. Fourth, add Fc mutations to decrease ADC catabolism in healthy tissue due to normal antibody catabolism pathways leading to extended systemic exposure. Fifth, insert an unpaired cysteine in a shielded location to enable site-specific DAR2 ADC generation yielding an ADC with reduced hydrophobicity profile.

### Identification of S375C for site-specific conjugation and generation of a homogeneous ADC

We selected serine 375 in the human IgG1 heavy chain CH3 domain to convert to a cysteine residue (S375C, as defined by EU numbering format) to enable site-specific conjugation. The S375C mutation maintains key antibody functions such as homodimer formation, N-glycosylation, and binding to neonatal Fc receptor (FcRn), FcγR, and protein A based on literature (30–33) and does not affect developability (e.g. overall biophysical properties and hydrophobicity profile (internal data not shown). The S375 site is modeled to be shielded and sequestered from interactions with solvent (i.e. a low (<30%) solvent accessible surface area). Experimental structural insights confirmed the site to be optimal for shielding of the payload after conjugation (34). Payload attachment to the site minimally increases the hydrophobicity of the resulting ADC which is important given the high hydrophobicity of many cytotoxic payloads including MMAE. Reduced ADC hydrophobicity results in improved *in vitro* stability and reduced aggregation potential during storage. Importantly, lower ADC hydrophobicity profiles improve *in vivo* exposure, efficacy, and tolerability (35). Moreover, antibody aggregates have the potential risk of being internalized via FcγRs in a target-independent fashion (36, 37). When the term “sacituzumab-SL-MMAE” is used in this paper, it will be sacituzumab’s amino acid sequence with the single S375C mutation. Additional sacituzumab amino acid sequence modifications will be denoted within a parenthesis that follow sacituzumab’s name.

### Re-humanization of sacituzumab

TROP2-SL-MMAE’s antibody originated from the sacituzumab antibody component of the anti-TROP2 ADC sacituzumab govitecan (11). Sacituzumab’s sequence (38) was analyzed with BIOVIA Discovery Studio software package using the “Predict Humanizing Mutations” tool and three re-humanized sacituzumab variants were generated: BSM (Best Single Mutations), FRS (Frequent Residue Substitutions) and GS (Germline Substitutions). The BSM, GS, and FRS re-humanized sequences had a combination of eleven, ten, or nine mutations in the framework regions from both heavy and light chains, respectively. Sacituzumab and variants were produced and analyzed for affinity, titer, aggregation level, thermostability, and hydrophobicity. The variants’ affinity for human and rhesus TROP2 were comparable to sacituzumab (not shown). BSM notably had a lower hydrophobicity profile compared to sacituzumab and the other two variants by hydrophobic interaction chromatography (HIC) with a retention time (RT) = ~24 min versus 35 min (**Figure 1B** upper panel and **Figure 1C**). An improved hydrophobicity profile by reverse phase (RP) chromatography (**Figure 1B**, lower panels) was also seen with a decreased baseline post-main peak where the most hydrophobic species elute. In contrast, the original sacituzumab had elevated hydrophobicity by these assessments.

The BSM re-humanization approach was selected as the foundation to build the ADC candidate. BSM is a computational model that theoretically increases the stability of humanized antibody framework residues while maintaining human sequence identity. The sacituzumab variant generated was a summation of each individual point mutant in the framework region predicted to be stabilizing. The BSM variant containing eleven single mutations was calculated to provide -9.03 kcal/mol stabilization compared to the sacituzumab sequence as described in Materials and Methods. Additionally, the BSM sequence had higher identity (84% identity) to human germline IGHV7-4-1*02 and IGKV1-13*02 compared to sacituzumab (81% identity).

### Avidity tuning sacituzumab to bind TROP2-high cells with higher avidity over TROP2-low cells

In parallel with sacituzumab re-humanization, a yeast display campaign was performed to develop an avidity-tuned antibody that binds TROP2-high cells (>150K surface copy number) with higher avidity versus TROP2-low cells (<50K surface copy number) while simultaneously improving its biophysical properties. This range was chosen because human primary normal esophageal epithelial (Cell Biologics #H-6046), epidermal keratinocytes (ATCC #PCS-200-011), bronchial/tracheal epithelial (ATCC #PCS-300-010), and small airway epithelial cells (ATCC #PCS-301-010) expressed between 36K and 72K surface TROP2 copy number. In contrast, many TROP2 cancer cell lines expressed >200K surface TROP2 copy number. A site saturation mutagenesis yeast display library was generated using 61 VH and 43 VL single point mutant positions from sacituzumab’s CDR and framework. Amino acid substitutions were made at each position with each of the 17 amino acids (excluding methionine, tryptophan, and cysteine) for a total sequence variation of 976 VH and 731 VL mutant variants. Sacituzumab and its library of variants were displayed as Fabs on the surface of *Saccharomyces cerevisiae* (**Figure 2A**), and reduced affinity variants were selected by flow cytometry based on reduced binding to soluble recombinant human TROP2 compared to sacituzumab (**Figure 2B**). The final selection of 21 heavy chain (HC) and 20 light chain (LC) variants showing reduced affinities by up to 4 logs were analyzed for differential binding to human TROP2-high, human TROP2-low, and human TROP2-negative cells (**Supplementary Table 1**).

**Figure 2:**
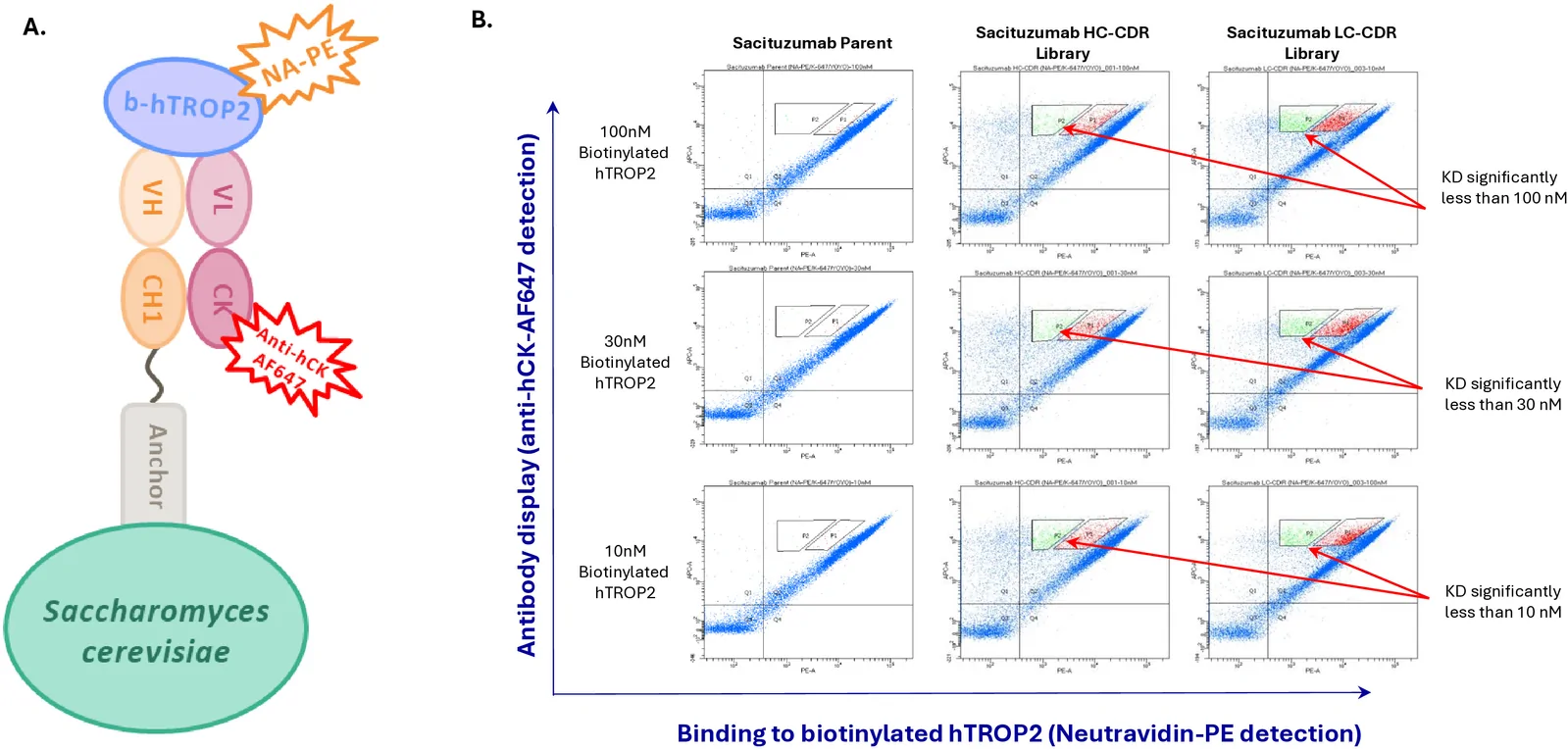
Sacituzumab avidity tuning using yeast display. **(A)** Schematic of yeast Fab display and binding to TROP2. Biotinylated TROP2 detected with neutravidin-PE (NA-PE) and yeast surface Fab density detected with AF647-labeled anti-human kappa antibody (anti-hCk-AF647) **(B)** Flow cytometry sorting of variants with reduced affinity to biotinylated TROP2. FACS plots and sorting gates for HC-CDR and LC-CDR libraries compared to parent sacituzumab antibody using 100 nM, 30 nM, and 10 nM biotinylated human TROP2.

Reduced affinity antibodies were ranked by their avidity potential to maintain equal or similar binding potency on TROP2-high cells (i.e. BxPC3; ~456K TROP2 molecules/cell) as sacituzumab while losing significant binding potency on TROP2-low cells (MDA-MB-231; ~43K TROP2 molecules/cell) compared to sacituzumab. TROP2-negative cells (mouse EMT6) were used to eliminate any mutant that displayed non-specific binding. Variants Y53D, Y105S, and F101E showed favorable differential binding behavior; whereas, Y92E and W106E showed significant reduction in binding to TROP2-high cells due to having greater affinity reduction (**Figure 3A**). The variants’ biophysical properties such as affinity by SPR, post-protein A aggregation by UP-SEC, purity by CE-SDS, thermostability by nano differential scanning fluorimetry (nano DSF; Tonset, the temperature at which the first conformational change start to occur; Tm1, the melting temperature at which 50% of the protein or most temperature-sensitive domain unfolds; Tagg, temperature of the onset of aggregation), and hydrophobicity by HIC column binding were assessed (**Supplementary Table 1**). **Figure 3B** shows a summary of variants (Y53D, F101E, Y105S) with optimal properties and less optimal properties (W106E, Y92E) as above described.

**Figure 3:**
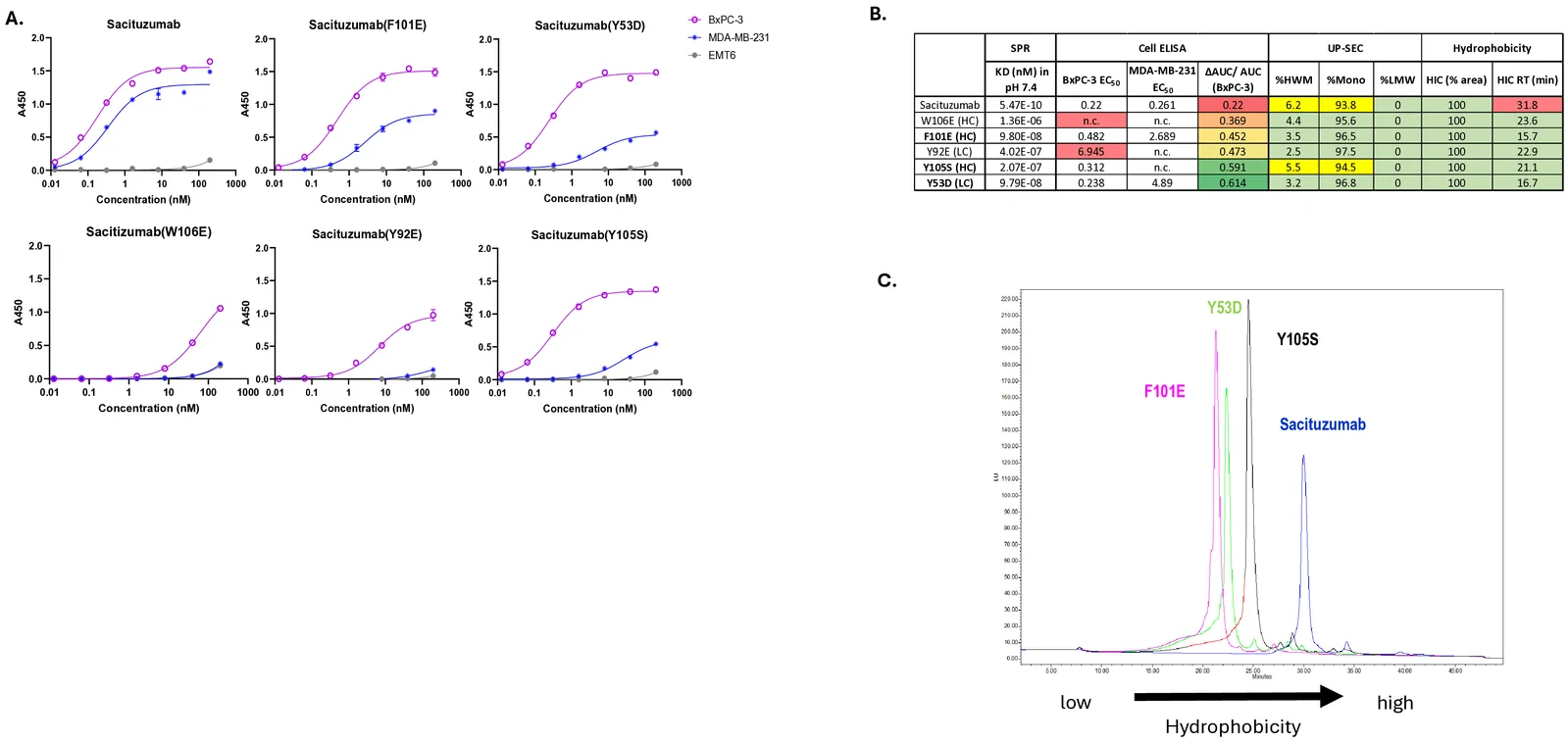
Identification of avidity-tuned sacituzumab variants with reduced hydrophobicity profile. **(A)** Cell binding ELISA of sacituzumab and selected variants to human TROP2-high cells (BxPC3), human TROP2-low cells (MDA-MB-231), and human TROP2-negative cells (mouse EMT6 cells). **(B)** Summary of sacituzumab and its avidity tuned variants’ properties: differential cell binding to cells lines with different TROP2 levels, aggregation measured by UP-SEC, thermostability (Tonset, Tm1, Tagg) measured by nano-DSF, and hydrophobicity measured by HIC using the Propac method/column. **(C)** HIC profile using Butyl method/column of sacituzumab and selected variants expressed in CHO cells and purified via multi-step purification.

Engineering and screening antibodies used for ADC generation towards lower hydrophobicity profiles early in the discovery phase increases the chance of identifying a developable ADC (39–44). Strong emphasis was placed on the selected variants’ hydrophobicity profile with an anticipation that improved exposure and a possible improved safety profile would result (35). Selected variants were produced by CHO transient transfection, purified via multi-step purification, and retention time (RT) analyzed using HIC-butyl columns. **Figure 3C** shows the HIC profiles for sacituzumab (RT~31.8 min) and some selected avidity-tuned variants: Y53D (RT~24 min), Y105S (RT~26 min), F101E (RT~23 min). The reported HIC-butyl RT differs from RT reported during screening stage (**Figure 3B**) due to use of different HIC column (HIC-propac). Variants were triaged based on affinity, differential binding to TROP2-low and TROP2-high cells, and key biophysical properties including hydrophobicity. Avidity-tuned sacituzumab molecules containing a LC mutation (Y53D) or HC mutation (F101E and Y105S) were constructed with the S375C modification in the Fc region to allow site-specific conjugation and generation of ADCs with the - SL-MMAE linker-payload.

### Evaluation of a panel of avidity-tuned variants for anti-tumor activity in the BxPC-3 pancreatic tumor xenograft model

Sacituzumab variants demonstrated potent in vitro cytotoxicity against a panel of TROP2+ cells that had TROP2 receptor density above 272K and lower potency on cells with lower TROP2 receptor density. Similar TROP2 receptor density impact on in vitro cytotoxicity potency was also seen with wildype sacituzumab ADC (**Figure 4A**). The avidity design goal was to achieve effective TROP2-based cytotoxicity in cells with a high TROP2 membrane density *in vivo* while decreasing cytotoxicity in cells that express lower TROP2 membrane density (mimicking normal tissue). The TROP2-high BxPC3 pancreatic xenograft model was used to compare the *in vivo* efficacy and pharmacokinetic profile of sacituzumab-MMAE with three avidity-tuned sacituzumab-SL-MMAE constructs. Y53D, F101E, and Y105S ADCs matched the efficacy of sacituzumab-SL-MMAE with similar ADC exposure (**Figures 4B, C**). Additionally, the levels of free MMAE released into the tumor by the three avidity-tuned variants was comparable to sacituzumab which aligned with their comparable efficacy (**Figure 4D**). The Y53D avidity-tuned variant was selected to move into an avidity-based mechanistic model based on its *in vivo* efficacy profile and that it had the best change in its cell binding characteristics and developability attributes (e.g. hydrophobicity) compared to other avidity-tuned options.

**Figure 4:**
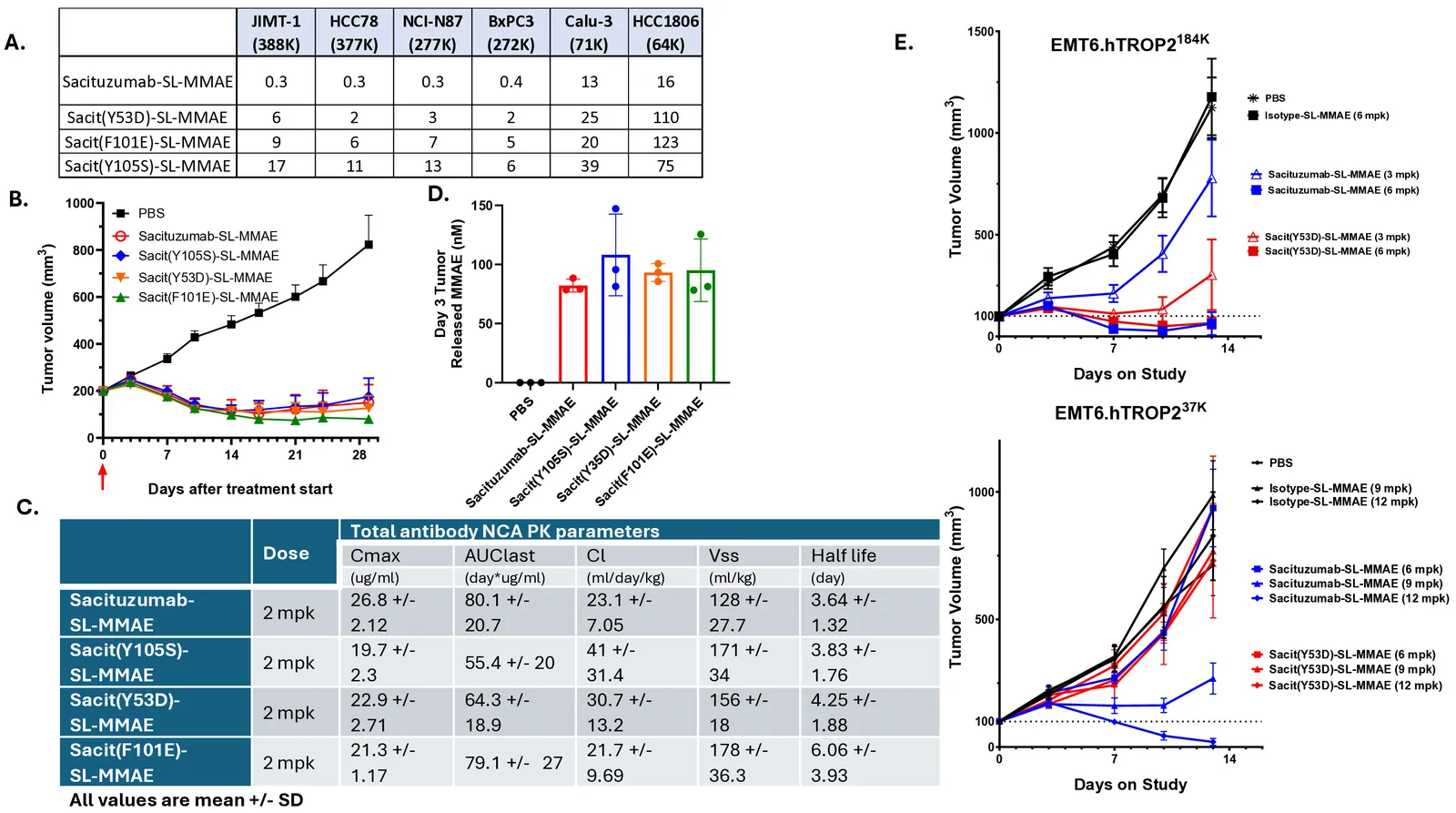
Evaluation of avidity-tuned sacituzumab variants for anti-tumor efficacy. **(A)** *In vitro* cytotoxicity potency evaluated on a panel of TROP2+ cells with differing receptor density with sacituzumab and prioritized avidity-tuned sacituzumab variants conjugated to SL-MMAE linker-payload. **(B)** *In vivo* anti-tumor efficacy assessed in BxPC3 tumor bearing mice by sacituzumab and prioritized avidity-tuned sacituzumab variants conjugated to SL-MMAE linker-payload. Sub-optimal 2 mg/kg single dose used to identify any avidity-tuned variant inferior to sacituzumab. **(C)** PK parameters of all test articles used in panel **(B)**. **(D)** Sacituzumab and prioritized avidity-tuned sacituzumab variants evaluated to distribute to and release free MMAE within tumor at Day 3 post-dosing. **(E)** Dose-dependent anti-tumor efficacy by sacituzumab-SL-MMAE and sacituzumab(Y53D)-SL-MMAE in mouse syngeneic EMT6 cell line that expressed human TROP2 at higher levels (184K receptors/cell) or lower levels (37K receptors/cell). 3 and 6 mg/kg doses were used for EMT6.TROP2^184K^ model system; 6, 9, and 12 mg/kg doses used for EMT6.TROP2^37K^ model system.

### Evaluation of avidity-tuning hypothesis to preferentially target *in vivo* TROP2-high cells over TROP2-low cells

A central hypothesis was that an avidity-tuned ADC would demonstrate TROP2 membrane density selective *in vivo* cytotoxicity, providing equivalent efficacy as high-affinity sacituzumab on tumor tissue while limiting binding and internalization on normal healthy tissues. Different membrane TROP2 densities were introduced into a TROP2-negative cell line to evaluate this hypothesis. Human TROP2 was introduced into the mouse syngeneic EMT6 cell line and individual clones were derived with differing TROP2 membrane density (TROP2 density = 37K, 66K, 184K, and ~4900K per cell). The mouse EMT6 model was chosen over other potential mouse syngeneic models for this question due to its inherent sub-nM cytotoxicity sensitivity to MMAE. The four clones and parental EMT6 cells were implanted subcutaneously into immune-competent Balb/c mice and tumors allowed grow for seven days (small ~100--200 mm^3^ tumors that would meet the inclusion criteria for an efficacy study) or twenty days (large tumors approaching the final day of efficacy study) before tumors were harvested to evaluate TROP2 expression by immunohistochemistry (IHC). Clones with increasing TROP2 membrane density *in vitro* generated tumors with increasing IHC staining intensity *in vivo*; each clone’s IHC staining intensity was similar at D7 and D20 post-implantation (i.e. minimal to no antigen loss over the time course of the study in immune-competent Balb/c mice).

TROP2-low EMT6.hTROP2^37K^ and TROP2-high EMT6.hTROP2^184K^ were chosen to assess the impact of TROP2 membrane -density *in vivo* cytotoxicity of sacituzumab(Y53D) compared to sacituzumab (both constructs used S375C for site-specific DAR2 conjugation of SL-MMAE). Doses of 3 and 6 mg/kg sacituzumab(Y53D)-SL-MMAE matched the anti-tumor efficacy of 3 and 6 mg/kg sacituzumab-SL-MMAE in the TROP2-high EMT6.hTROP2^184K^ model consistent with their equivalent efficacy in the TROP2-high BxPC3 human xenograft model. It was assumed that higher therapeutic doses yielding higher receptor occupancy would be needed in the TROP2-low EMT6.hTROP2^37K^ model system to deliver similar intracellular payload concentrations and elicit similar anti-tumor efficacy magnitude. As expected, 6 mg/kg sacituzumab-MMAE which had been efficacious in TROP2-high EMT6.hTROP2^184K^ model had minimal activity in the TROP2-low EMT6.hTROP2^37K^ model; 12 mg/kg sacituzumab-MMAE was needed to show robust efficacy in the TROP2-low EMT6.hTROP2^37K^ model. In alignment with the hypothesis, 12 mg/kg sacituzumab(Y53D)-SL-MMAE demonstrated minimal cytotoxicity in the TROP2-low EMT6.hTROP2^37K^ model as anticipated for an avidity-tuned antibody that inefficiently bound low TROP2+ cells. The TROP2-SL-MMAE variants demonstrate that the efficacious dose was dependent on the TROP2 membrane density. Moreover, sacituzumab (Y53D)-SL-MMAE ADC efficacy was more dependent on TROP2 expression level than the high-affinity sacituzumab-SL-MMAE ADC. For example, increasing the sacituzumab-SL-MMAE dose by two-fold (from 6 to 12 mpk) enabled potent efficacy on TROP2-low EMT6.hTROP2^37K^ model; whereas, the dose increase needed for sacituzumab(Y53D)-SL-MMAE to be efficacious on the Trop2-low cells, if any, was outside the range tested (**Figure 4E**).

### Evaluation of BSM and avidity-tuned variant in NHP PK studies

Incorporating BSM mutations to enhance sacituzumab’s humanness and hydrophobicity profile did not affect its pharmacokinetic (PK) profiles in rhesus monkeys as evidenced by the overlapping PK profiles of the sacituzumab(BSM) compared to sacituzumab following 3 mg/kg intravenous administration (**Figure 5A**). The addition of Y53D mutation to sacituzumab(BSM), which further reduced its hydrophobicity and avidity-tuned the interaction with TROP2 in normal tissues, demonstrated increased plasma exposure compared to the non-Y53D variant following 1 mg/kg intravenous administration (**Figure 5B**). Additionally, sacituzumab exhibited moderate TMDD at doses ranging from 0.1 mg/kg to 3 mg/kg (**Supplementary Figure 2**). The Y53D mutation reduced the TMDD and exhibited linear PK in the dose range of 1 mg/kg to 20 mg/kg (**Figure 5B**). Y53D was selected as avidity-tuned variant to build an ADC based on its improved biophysical properties, *in vitro* TROP2 receptor density binding differential, and *in vivo* TROP2 receptor density efficacy profile.

**Figure 5:**
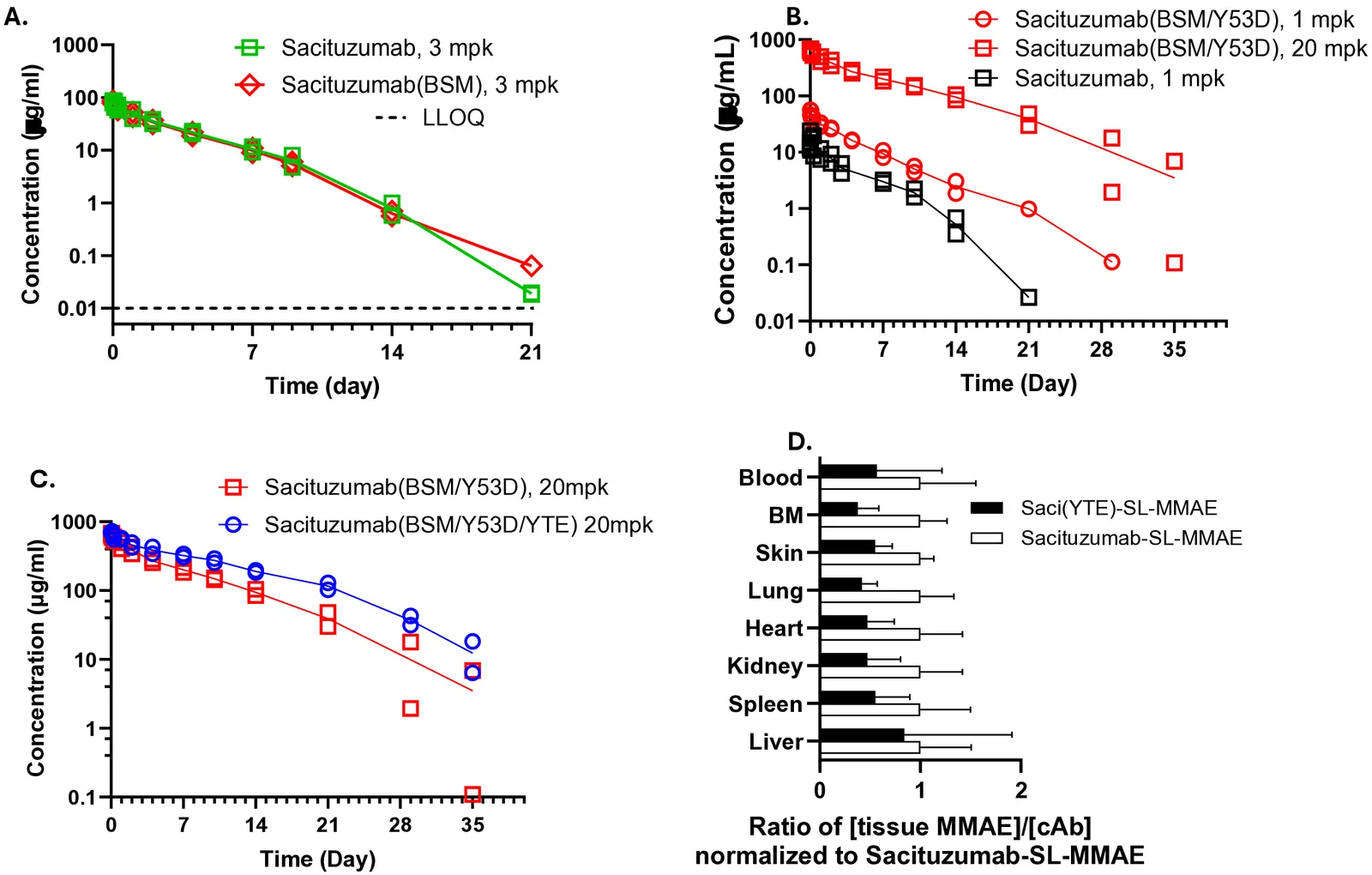
PK of sacituzumab and its engineered variants in rhesus non-human primate. **(A)** PK of sacituzumab and sacituzumab(BSM) in individual rhesus monkeys after a single IV administration of 3 mg/kg. **(B)** PK of sacituzumab at 1 mg/kg and sacituzumab(BSM/Y53D) at either 1 or 20 mg/kg in individual rhesus monkeys. **(C)** PK of sacituzumab(BSM/Y53D) and sacituzumab(BSM/Y53D/YTE) at 20 mg/kg in individual rhesus monkeys. LLOQ: lower limit of quantitation. **(D)** Tissue MMAE concentrations harvested fourteen days after administration of 30 mg/kg Sacituzumab-SL-MMAE or Saci(YTE)-SL-MMAE into Tg32 mice that express human FcRn. The ratio of day 14 tissue MMAE concentrations to day 14 cAb levels were normalized sacituzumab-SL-MMAE ratio.

### Incorporation of the YTE mutation to minimize normal tissue catabolism of the ADC

ADC clinical development has a high failure rate due to toxicity limiting the tolerated ADC doses to levels below those required for substantial anti-tumor efficacy. Dose-limiting toxicities are commonly associated with cells and tissues that do not express the targeted antigen (*i.e.* off-target toxicity) due to the non-specific cellular uptake and catabolism of the ADCs (45). FcRn and its pH-dependent interactions with antibodies represent one of the most important salvage mechanisms in modulating antibody clearance in the endosomal compartment leading to improved antibody recycling, minimized normal tissue catabolism, and extended half-life (46). The YTE mutation (M252Y, S254T, and T256E) engineered into the CH2 domain of the Fc region has been a successful strategy to enhance the FcRn binding profile and optimize the PK properties of non-ADC antibodies (47, 48). Incorporation of YTE was explored to determine if the systemic normal tissue catabolism of sacituzumab(BSM/Y53D) could be further reduced in Tg32 mice which express human FcRn and in NHP. After a single IV administration in NHP, at 20 mg/kg, sacituzumab(BSM/Y53D)/-YTE showed improved exposure compared to sacituzumab(BSM/Y53D) with AUC increased by approximately 1.7 fold (**Figure 5C**) supporting reduced normal tissue catabolism. Tg32 mice were used to evaluate if normal tissue catabolism of the ADC would be impacted by incorporating YTE into the ADC. Saci(YTE)-SL-MMAE plasma exposure (AUClast= 2890 ug*d/ml) was higher than that of sacituzumab-SL-MMAE (AUClast=2,210 ug*d/ml), presumably due to improved recycling mediated by the YTE mutations and enhanced human FcRn interactions. As anticipated, the ratio of MMAE normal tissue delivery to plasma ADC exposure was lower for Saci(YTE)-SL-MMAE than for Sacituzumab-SL-MMAE (**Figure 5D**). These data support the rationale for incorporating on YTE into sacituzumab(BSM/Y53D) to potentially reduce normal tissue catabolism and the associated risk of increased toxicity.

TroptimAb exhibited a reduced hydrophobicity compared to unconjugated sacituzumab (**Figure 6A**). For comparison, sacituzumab elutes with a HIC RT ~31 min and its corresponding MMAE ADC with a RT ~37 min showing that TroptimAb-SL-MMAE reduced hydrophobicity profile by using BSM and Y53D mutations successfully led to a DAR2 MMAE ADC with acceptable hydrophobicity profile (**Figure 6B**).

**Figure 6:**
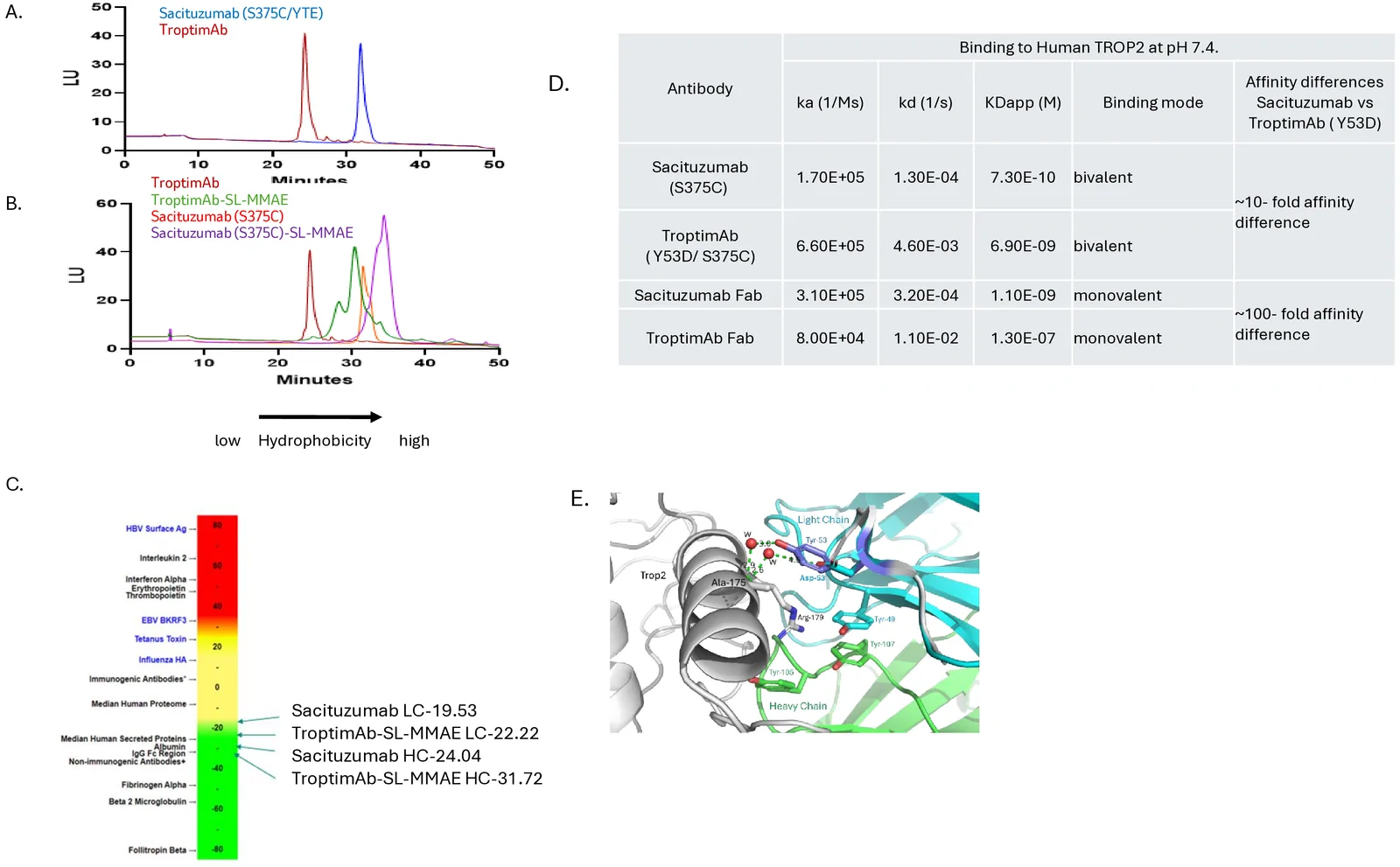
TroptimAb-SL-MMAE characterization: HIC Elution profile of **(A)** TroptimAb-SL-MMAE and sacituzumab (S375C/YTE), **(B)** TroptimAb-SL-MMAE, TroptimAb sacituzumab (S375C) antibody, and sacituzumab (S375C)- SL-MMAE ADC using HIC (butyl method **C**) *In silico* immunogenicity risk assessment of TroptimAb-SL-MMAE’s and sacituzumab’s heavy chains and light chains **D**) Affinity to human TROP2 at pH 7.4 measured by SPR in bivalent format (with mAbs and ADC) and monovalent format (with Fabs) **(E)** Structure of the TroptimAb/TROP2 interaction network by x-ray crystallography).

An *in silico* immunogenicity risk assessment of amino acid sequences of sacituzumab and the optimized sacituzumab variant used in TroptimAb-SL-MMAE was performed using EpiMatrix v1.2 with DR9 software. The optimized antibody variant’s HC and LC amino acid sequences have lower epitope risk than the corresponding sequences in sacituzumab (**Figure 6C**). By SPR, Sacituzumab antibody and Fab were compared with TroptimAb and Fab using immobilized TROP2 to better characterize the bivalent versus monovalent affinity of TroptimAb-SL-MMAE. In a bivalent format, TroptimAb showed a 10-fold affinity difference compared to sacituzumab, while TroptimAb-SL-MMAE Fab showed a 100-fold affinity difference compared to sacituzumab Fab (**Figure 6D**). In summary, both TroptimAb and TroptimAb-SL-MMAE showed favorable biophysical and developability characteristics.

The crystal structures of the human TROP2 ectodomain bound to sacituzumab and TroptimAb Fabs were determined to evaluate how the Y53D substitution alters antigen engagement (**Figure 6E**). In the TroptimAb structure, two Fabs bind across a single TROP2 dimer in a 2:2 configuration, similar to the mode of engagement observed for sacituzumab (PDBid: 9PI9), and Fab binding induces a conformational rearrangement of the dimer relative to apo TROP2. The TROP2–TroptimAb complex reveals an extensive interaction network contributed by heavy chain CDR1 and CDR3 and -light chain CDR1–CDR3, with clear electron density for Y53D in light chain CDR2. In the sacituzumab complex, Y53 lies just outside the central paratope but participates in a conserved water mediated hydrogen bond with the TROP2 A174 backbone and engages in packing interactions with R178 (TROP2), Y49 (LC), Y107 (HC), and Y105 (HC). This structured water molecule is observed- in both the TroptimAb and sacituzumab complexes. Replacement of 53 (Y53D) with Asp disrupts the water mediated- hydrogen bond and destabilizes the local aromatic stacking network, providing a structural explanation for the reduced binding affinity (**Figure 6E**). While this manuscript was in preparation, Ferraro et al. reported a sacituzumab–TROP2 complex with the same 2:2 Fab:dimer stoichiometry and a corresponding conformational rearrangement of the TROP2 dimer. They further show by cryoEM- and SECMALS that the intact antibody can engage two TROP2 dimers in a 2:4 assembly, and they also identify the same conserved water molecule bridging Y53 and A-174. These findings support our observations that the Fab epitope spans the dimer proximal- surface and that antibody binding stabilizes a distinct TROP2 conformation—features that amplify the functional impact of perturbations within the L-CDR2 microenvironment at position 53 (49).

### TroptimAb-SL-MMAE characterization

TroptimAb-SL-MMAE binds to human (K_D_ = ~48 nM) and rhesus monkey (K_D_ = ~110 nM) TROP2. No binding was observed to canine, rat, nor mouse TROP2 at up to 1 uM TROP2 concentration **Supplementary Table 2**). Since soluble TROP2 was injected over captured TroptimAb-SL-MMAE; the reported apparent K_D_ may be due to bivalent interactions. There are two common single nucleotide polymorphisms leading to an amino acid change in TROP2’s extracellular domain: D173A and D216E. D173 sits on a well-defined helix that interacts with sacituzumab and TroptimAb; however, it does not directly interact with either antibody. D216 is located further away from the antibodies’ epitope. Sacituzumab and TroptimAb bound both somatic TROP2-His variants within two-fold the affinity they had to consensus TROP2-His (data not shown). TroptimAb-SL-MMAE’s specificity to human TROP2 was assessed by evaluating its affinity to human EpCAM which is the closest homolog to human TROP2. TroptimAb-SL-MMAE did not bind human EpCAM when tested up to 1 µM antigen (data not shown).

TroptimAb-SL-MMAE’s *in vitro* plasma stability was evaluated using plasma from C57BL/6 mouse, Wistar Hannover rat, rhesus monkey, and human (**Figure 7A**). MMAE release from 50 µg/mL TroptimAb-SL-MMAE increased progressively at 37 °C across the different species. The cumulative amount of MMAE released at day 7 in mouse, rat, monkey, and human plasma corresponded to approximately 2%, 0.3%, 0.5%, and 0.5% of the total MMAE that was originally conjugated to TroptimAb-SL-MMAE, respectively. Alternatively, intact mass of TroptimAb-SL-MMAE from the *in vitro* plasma stability samples was used to calculate average DAR over incubation time (**Figure 7B**). The change in DAR over time was similar across all four species with the maximum decrease in DAR being less than 15% after 7 days. These results indicated minimum release of MMAE in plasma from preclinical species and humans.

**Figure 7:**
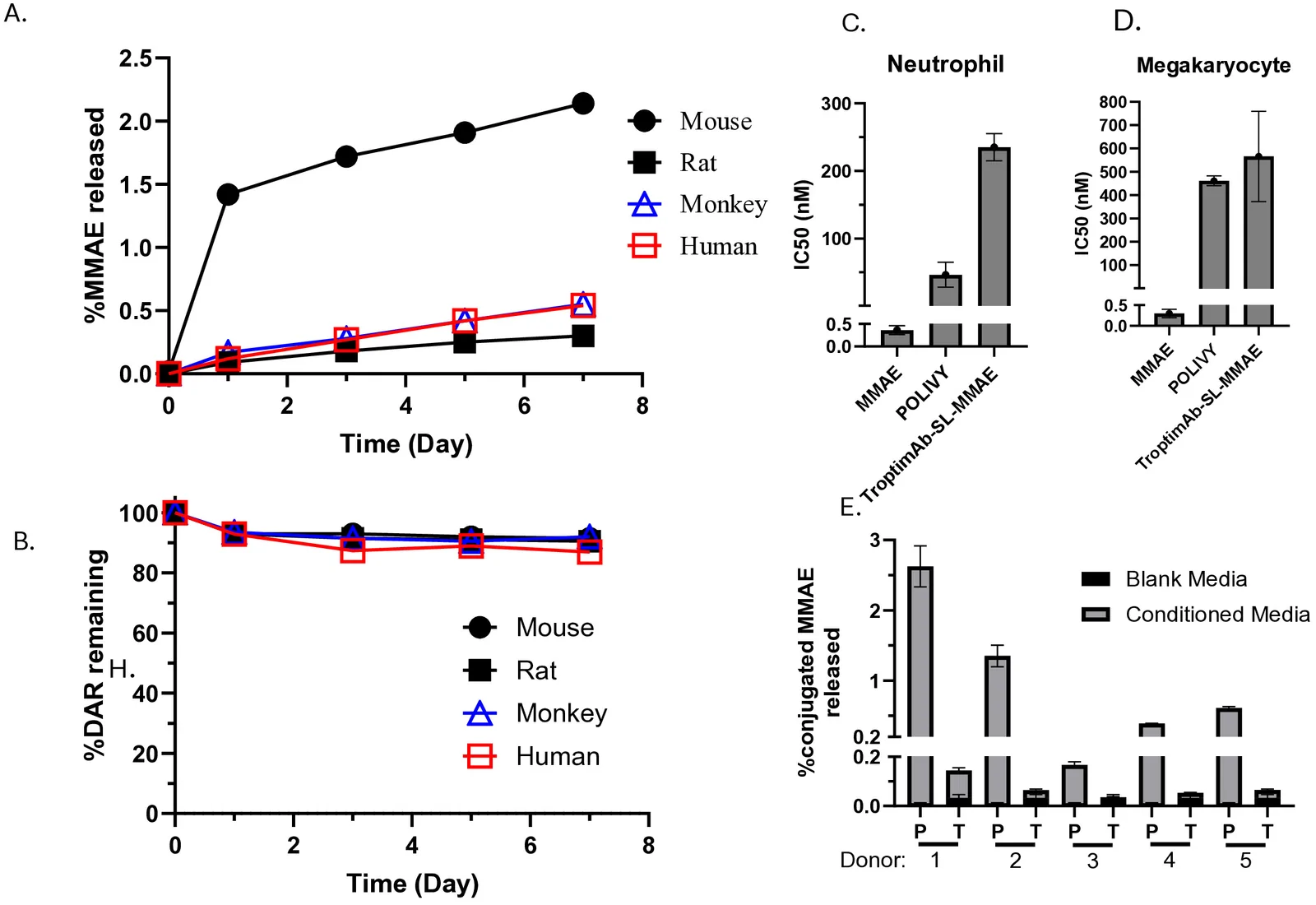
**(A)** %MMAE released after incubating TroptimAb-SL-MMAE in C57BL/6 mouse, Wistar Hannover rat, rhesus monkey, and human plasma at 37 °C for seven days. **(B)** % TroptimAb-SL-MMAE DAR remaining after incubating in C57BL/6 mouse, Wistar Hannover rat, rhesus monkey, and human plasma at 37 °C for seven days. **(C)** *In vitro* cytotoxicity of human neutrophils being differentiated from human hematopoietic stem cells. Average results from four donors combined. **(D)** *In vitro* cytotoxicity of human megakaryocytes being differentiated from human hematopoietic stem cells Average results from four donors combined. **(E)** Release of free MMAE from POLIVY^®^ and TroptimAb-SL-MMAE following incubation with basal media or conditioned media from neutrophil differentiation cultures harvested from five donors (P=POLIVY^®^, T=TroptimAb).

TroptimAb-SL-MMAE’s *in vitro* stability and off-target uptake potential was also evaluated using an *in vitro* model system where human hematopoietic stem cells were differentiated into neutrophils or megakaryocytes (50, 51) (**Figures 7C, D**); TroptimAb-SL-MMAE’s off-target cytotoxicity potency was compared to an approved MMAE ADC that carries warning for myelosuppression (POLIVY^®^, polatuzumab vedotin) (22). TroptimAb-SL-MMAE (DAR2) was ~5-fold less potent (on a molar conjugated-MMAE basis), than POLIVY^®^ (DAR~4) in the neutrophil cytotoxicity assay (IC_50_ = 240 +/- 20 nM (n=3) versus 50 +/- 20 nM (n=3), respectively) (**Figure 7C**). Both TroptimAb-SL-MMAE and POLIVY^®^ had similar off-target potency in the megakaryocyte cytotoxicity assay (IC_50_ = 600 +/- 200 nM (n=3) versus 460 +/- 20 nM (n=3), respectively) (**Figure 7D**). The reduced off-target cytotoxicity potency of TroptimAb-SL-MMAE compared to POLIVY^®^ in the neutrophil assay was further explored by modifying the assay (50). Neutrophil conditioned media (containing secreted neutrophil proteolytic enzymes) or basal media was spiked with 667 nM of either ADC (100 ug/ml) and the amount of free MMAE liberated quantified. Upon exposure to neutrophil conditioned media, ~1% (range 0.15%-2.6%) of the conjugated MMAE from DAR4 POLIVY^®^ was released compared to ~0.04% (range 0.002%-0.1%) of the conjugated MMAE was released from DAR2 TroptimAb-SL-MMAE. The reduced MMAE levels liberated from TroptimAb-SL-MMAE (**Figure 7E**) maybe due to the higher stability of the linker connection to the antibody and the lower protease accessibility to the linker conjugation site. TroptimAb-SL-MMAE’s S375C engineered site was chosen, in part, because it is buried within the Fc CH3 domain, while POLIVY^®^’s intrachain cysteines are in the Fab region which may be more exposed and vulnerable to protease activity. Alternatively, the different protease cleavage sequence chosen may have contributed to the different outcome.

### TroptimAb-SL-MMAE *in vitro* cytotoxicity and *in vivo* efficacy profile using cell lines

TroptimAb-SL-MMAEs *in vitro* cytotoxicity was evaluated using human cancer cell lines and normal primary cells expressing a range of surface TROP2 densities (**Figure 8A**). Eight human cancer and two normal primary cell lines were incubated with a dose titration of TroptimAb-SL-MMAE for four days and cytotoxicity measured; isotype-MMAE (DAR2) and unconjugated free MMAE were included as controls. TroptimAb-SL-MMAE demonstrated more potent cytotoxicity in TROP2-high cancer cells (JIMT-1, HCC78, NCI-N87, BxPC3) than to cancer cells that express moderate (Calu-3, AGS) to low (HCC1806, MDA-MB-231) TROP2. Primary keratinocytes (PCS-200) and lung small airway epithelial cells (PCS-301) that expressed low TROP2 levels in culture were less sensitive to TroptimAb-SL-MMAE than some TROP2-high cancer cell lines. TroptimAb-SL-MMAE activity on SW620 cancer cells was comparable to isotype-SL-MMAE, as expected, given that SW620 cells did not express TROP2 (data not shown). Free MMAE was potent in all cell lines demonstrating that cell lines used were responsive to the cytotoxic payload in the absence of TROP2-targeted delivery.

**Figure 8:**
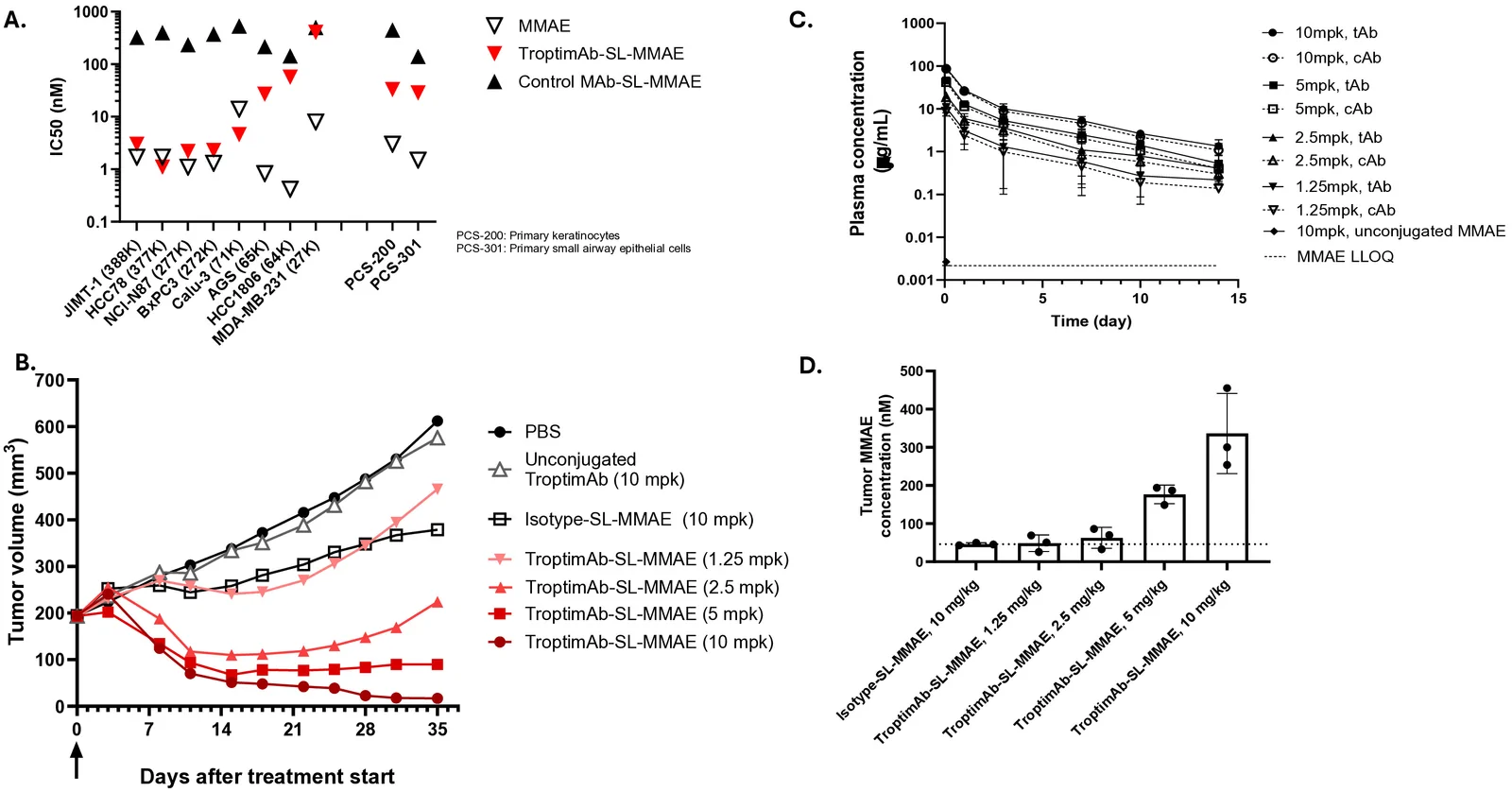
TroptimAb-SL-MMAE dose-dependent anti-tumor efficacy in the BxPC-3 pancreatic tumor xenograft model. **(A)** TroptimAb-SL-MMAE cytotoxicity potency using panel of cell lines with different TROP2 receptor densities; isotype-MMAE and free MMAE included as controls. Cytotoxicity assessed using CTG assay and IC50 values denoted. **(B)** BALB/c nude mice implanted with BxPC-3 cells subQ received IV administration of a single dose at Day 0 of PBS, isotype control antibody-MMAE (Isotype-SL-MMAE), unconjugated TroptimAb, or TroptimAb-SL-MMAE at 1.25, 2.5, 5, or 10 mg/kg. Statistically significant tumor regression was observed in the groups treated with TroptimAb-SL-MMAE at 2.5 mg/kg (p<0.05), 5 mg/kg, (p<0.01), or 10 mg/kg (p<0.01) compared with the control PBS group. Treatment with Isotype-SL-MMAE, the unconjugated TroptimAb, and 1.25 mg/kg TroptimAb-SL-MMAE did not show significant tumor regression (p>0.05)-. **(C)** Pharmacokinetic profile of MMAE-conjugated antibody (cAb), total antibody (TAb); and free MMAE analyzed. The mean plasma concentrations for each treatment group and standard deviation (N = 5/group from the efficacy cohorts) shown. MMAE LLOQ = 0.00215 μg/ml. **(D)** The mean tumor MMAE concentrations and standard deviation (N = 3/group from satellite terminal groups). The molar concentration calculated assuming 1 g tissue is equivalent to 1 mL in volume.

TroptimAb-SL-MMAE demonstrated dose-dependent anti-tumor activity in the BxPC-3 xenograft model (**Figure 8B**). Statistically significant tumor regression was observed in the groups treated with TroptimAb-SL-MMAE at 2.5 mg/kg (p<0.05), 5 mg/kg (p<0.01), and 10 mg/kg (p<0.01) compared to the control PBS group. Minimal tumor growth inhibition (TGI) was observed with 1.25 mg/kg TroptimAb-SL-MMAE and 10 mg/kg anti-RSV-SL-MMAE treatment. Unconjugated TroptimAb did not elicit anti-tumor activity indicating that there was minimal antibody-dependent cytotoxicity and complement-dependent cytotoxicity contribution to TroptimAb-SL-MMAE’s anti-tumor activity in this model. The mean percent body weight change of all treatment groups was comparable to the control PBS group suggesting that treatment with TroptimAb-SL-MMAE was tolerated [data not shown]. Since TroptimAb-SL-MMAE does not bind to mouse TROP2, its tolerability profile in mice does not address TroptimAb-SL-MMAE’s reduced TROP2-mediated toxicity hypothesis. The area under the concentration-time curve (AUC) and maximum concentration (Cmax) for both TroptimAb-SL-MMAE’s total antibody (TAb) and conjugated antibody (cAb) increased approximately proportionally from 1.25 to 10 mg/kg; PK parameter summary is in **Supplementary Table 3**. TAb and cAb exposure were overlapping suggesting that the TroptimAb-SL-MMAE linker was stable at the tested doses within the study duration (**Figure 8C**). Unconjugated MMAE in the plasma was mostly below limit of quantification and was only detectable at the early time point in the 10 mg/kg dosing group. The tumor MMAE concentrations three days after TroptimAb-SL-MMAE dosing showed approximately dose-proportional increase (**Figure 8D**). TroptimAb-SL-MMAE delivered more than seven-fold more MMAE into TROP2 expressing BxPC3 tumors than the isotype control ADC at the same 10 mg/kg dose.

The downstream cellular mechanisms responsible for MMAE-mediated tumor killing were explored. Since MMAE is a microtubule inhibitor, it is expected to induce G2/M arrest and apoptosis. Histone H3 is phosphorylated (pHH3) during chromosome condensation as cells enters the late G_2_ and M phases of cell cycle. MMAE delivered into the cytoplasm of a target cell bind tubulin disrupting mitotic spindle dynamics to cause G2/M arrest and prevent the cell from completing division. Therefore, an effective MMAE ADC therapeutic will lead to an accumulation of tumor cells still in mitosis with a resulting increase in number of pHH3-positive cells. BxPC3 tumors were dosed with increasing TroptimAb-SL-MMAE and tumors harvested at various timepoints to evaluate the time-course of initiating mitotic dysfunction as assessed by an increase in pHH3 signal. Alteration in the basal pHH3 signal was seen at the first timepoint (8 hrs post-dose) with maximal signal at day 3. The elevated pHH3 signal had mostly returned to baseline by day 10 (**Supplementary Figure 3A**). Apoptosis induction by MMAE is associated with the activation of caspases such as the cleavage of caspase 3 (CC3). The time-course for initiating apoptosis was assessed by the increase in tumor CC3 signal and compared to the timecourse of pHH3 induction. The elevated CC3 peaked at day 3 and resolved by day 10 with no clear temporal separation between the pHH3 timecourse and the CC3 timecourse (**Supplementary Figure 3B**). Following IV dosing, TroptimAb-SL-MMAE quickly distributed into the tumor mass causing mitotic dysfunction and apoptosis induction within hours and peaking by day 3.

### TroptimAb-SL-MMAE *in vivo* efficacy profile in human PDX models

Anti-tumor activity was evaluated in a panel PDX model spanning different indications and different TROP2 expression levels (TROP2 H-score in figure legend): BL5003 (bladder cancer), BL9214 (bladder cancer), BR1282 (triple negative breast cancer, invasive ductal carcinoma), BR1458 (triple negative breast cancer, invasive ductal carcinoma), LU0861 (non-small cell lung cancer (NSCLC), squamous cell carcinoma), LU0387 (NSCLC, adenocarcinoma), LU11694 (NSCLC, adenocarcinoma), and LU2049 (NSCLC, adenocarcinoma, NOS). Tumor bearing animals were administered TroptimAb-SL-MMAE or isotype MAb-SL-MMAE at 10 mg/kg on Days 0 and 21; a group of animals were treated with PBS as a control. Untreated tumor samples from the PDX tumor models revealed a range of TROP2 membrane expression by IHC [**Figure 9B**]. TroptimAb-SL-MMAE showed significant anti-tumor activity against TROP2 membrane high breast model BR1282 (p<0.01), bladder model BL5003 (p<0.01) and lung models LU0861, LU0387, and LU2049 (all p<0.01) compared with the control PBS group (**Figure 9A**). Bladder model BL9214 (weaker TROP2 membrane expression) and breast model BR1458 (undetectable TROP2 membrane expression) were resistant to TroptimAb-SL-MMAE activity consistent with TroptimAb-SL-MMAE target-dependent cytotoxic mechanism of action. It was notable that lung model LU11694 which had high TROP2 membrane expression was resistant to TroptimAb-SL-MMAE activity supporting TROP2 membrane expression is required, but not sufficient to potently kill TROP2+ PDX models. It was notable that LU11694 had the highest ABCB1/Pgp efflux pump baseline expression amongst all the PDX models in the panel; ABCB1 is a known MMAE resistance mechanism (**Supplementary Figure 4A**).Minimal TGI was observed with isotype-SL-MMAE compared with PBS group. TroptimAb-SL-MMAE (DAR~2; 10 mpk q3Wx2) efficacy was compared to two approved TROP2-Top1 ADC, Dato-DXd (DAR~4; 10 mpk q3Wx2) and TRODELVY/sacituzumab govitecan (DAR~8; 20 mpk BIWx4), in the eight PDX models. Similar efficacy was obtained with TroptimAb-SL-MMAE with either a lower payload dose (Dato-DXd; DAR~4; 10 mpk q3Wx2) or a much lower payload dose (TRODELVY; DAR~8; 20 mpk BIWx4) (**Supplementary Figure 4B**). Three additional PDX models (LU2505 (NSCLC), BR2014 (TNBC), and BR5400 (TNBC)) were sensitive to isotype-SL-MMAE which precluded making conclusions on role of TROP2 targeting in those models (data not shown). The mean %body weight change of the TroptimAb-SL-MMAE treated group was comparable to the control PBS group suggesting that treatment with TroptimAb-SL-MMAE was tolerated at the doses administered (data not shown).

**Figure 9:**
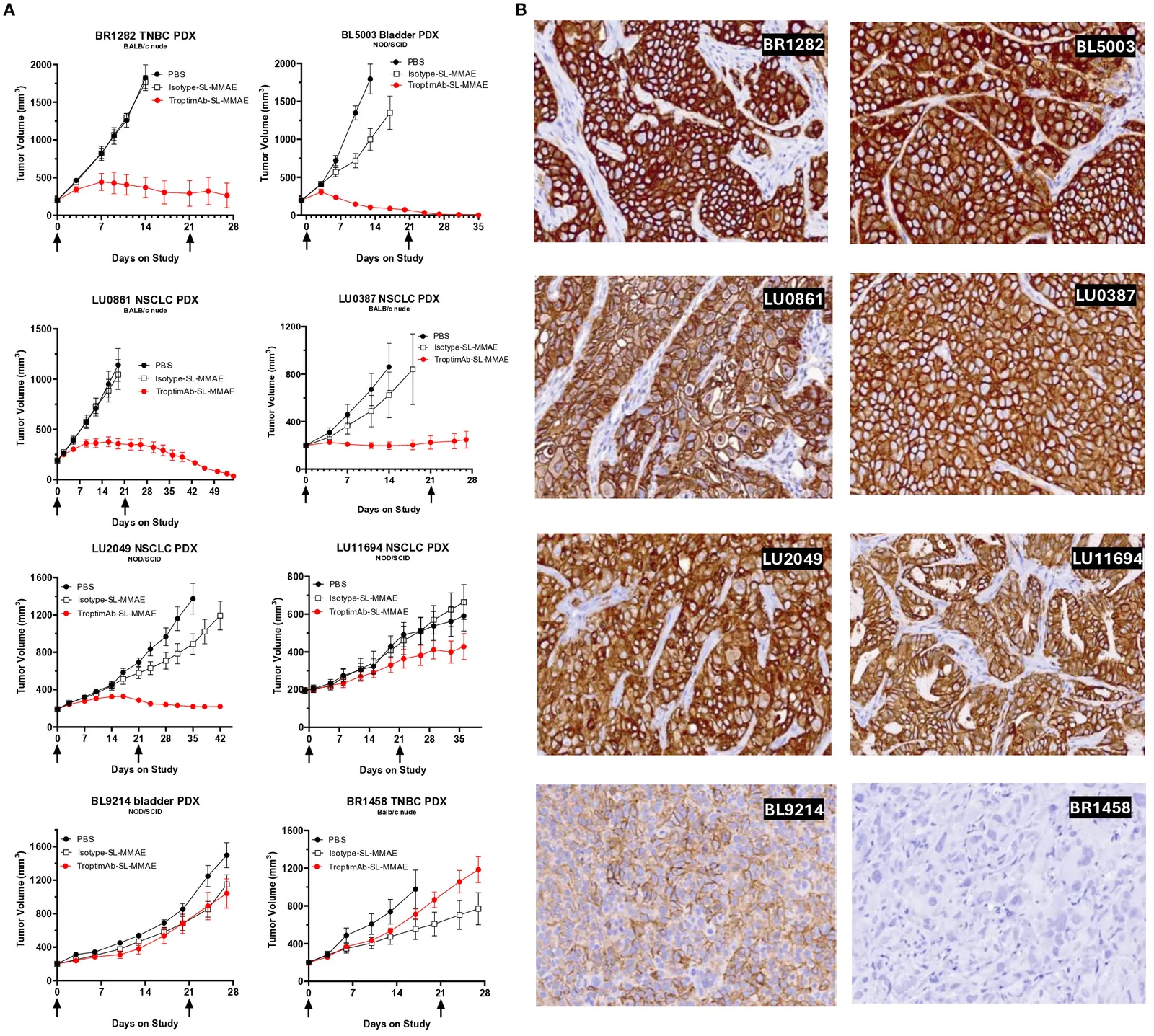
TroptimAb-SL-MMAE anti-tumor efficacy in PDX models. **(A)** PDX models established in immunodeficient BALB/c nude or NOD/SCID mice and treated with IV administration of 2 doses of PBS or 10 mpk TroptimAb-SL-MMAE or isotype-SL-MMAE (n=5 mice per treatment group). Arrows denote treatment at Days 0 and 21. Statistically significant tumor regression was observed in 5 of 8 models treated with TroptimAb-SL-MMAE at 10 mpk (p<0.01) compared with the control PBS group that correlated with **(B)** robust TROP2 membrane protein expression by IHC. Treatment with 10 mpk isotype-SL-MMAE did not show robust tumor regression in these models. PDX TROP2 H-score (0-300): BR1282 TNBC (300), BL5003 bladder (300), LU0861 NSCLC (300), LU0387 NSCLC (300), LU2049 NSCLC (300), LU11694 NSCLC (300), BL9214 bladder (228), BR1458 TNBC (0).

### Efficacy of TroptimAb-SL-MMAE in combination with PD-1 blockade in an engineered TROP2 syngeneic mouse model

The anti-tumor activity of TroptimAb-SL-MMAE as a single agent or in combination with anti-mouse PD-1 muDX400 in the syngeneic EMT6.hTROP2^184K^ tumor model was assessed. Combination treatment with 3 mg/kg TroptimAb-MMAE and 5 mg/kg muDX400 showed greater anti-tumor benefit than 3 mg/kg TroptimAb-SL-MMAE as a single agent. Overall, there were nine mice in the “ TroptimAb-SL-MMAE and muDX400 combination” group determined to have complete tumor regression after consecutive measurements of no measurable tumor. Approximately nine weeks post-tumor regression, all tumor-free mice were rechallenged with EMT6.hTROP2^184K^ cells. Eight of the nine mice with complete tumor regression in the “TroptimAb-SL-MMAE- and muDX400 combination” group remained tumor-free at day 47 post-rechallenge. All ten control naïve mice reached the tumor burden limit by day 28 post-inoculation (**Figure 10**). The mean %body weight change of mice treated with TroptimAb-SL-MMAE as a single agent or in combination with muDX400 was less than 1% following TroptimAb-SL-MMAE administration suggesting that treatment with TroptimAb-SL-MMAE was well tolerated (data not shown).

**Figure 10:**
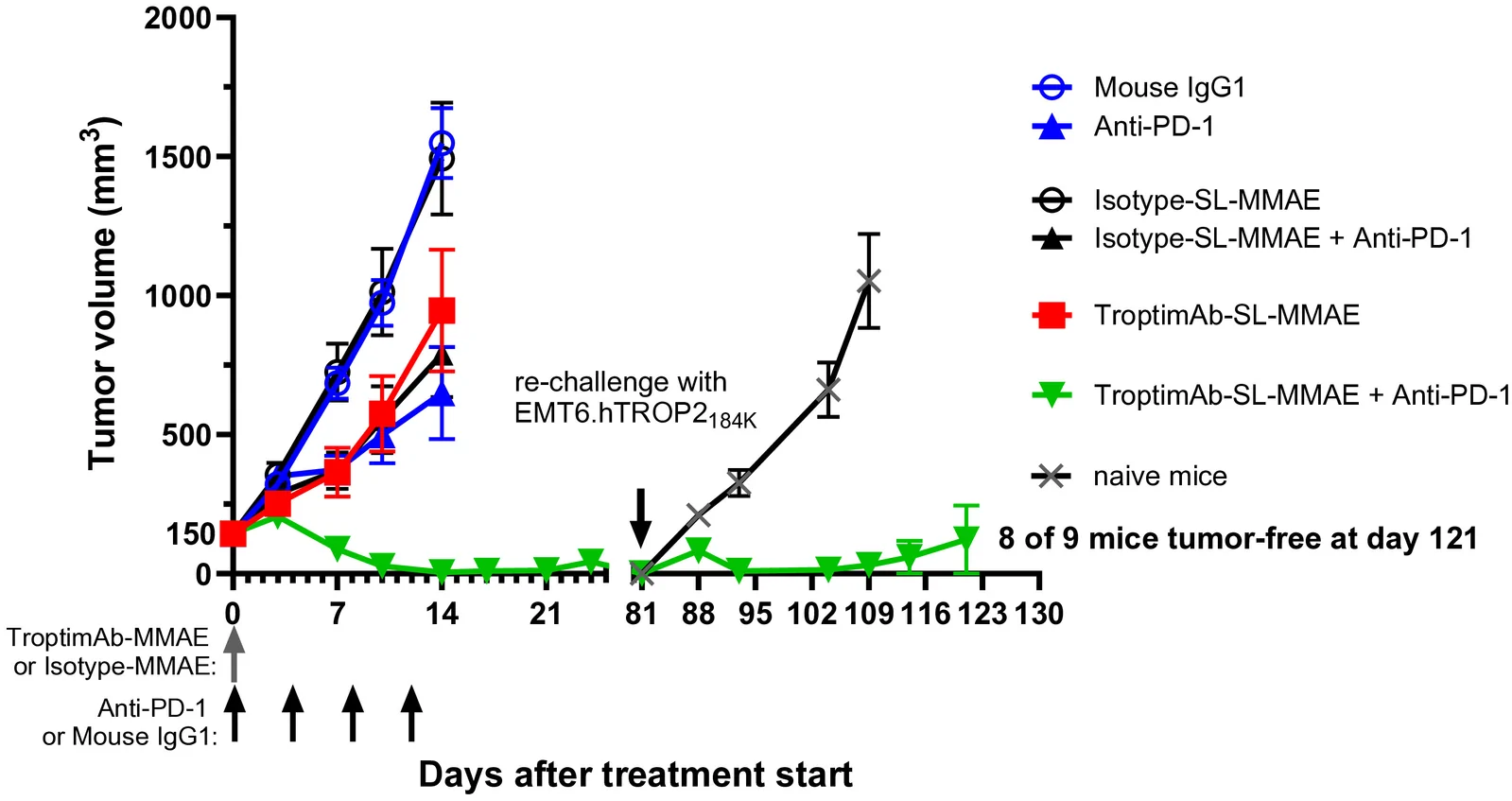
TroptimAb-SL-MMAE combination anti-tumor efficacy with anti-PD-1 in the syngeneic mouse EMT6.hTROP2^184K^ tumor model. Immunocompetent BALB/c mice were subcutaneously implanted with mouse EMT6 cells engineered to express hTROP2 (EMT6.hTROP2^184K^) and received IV administration of different combinations of TroptimAb-SL-MMAE, isotype-SL-MMAE, anti-PD-1, or mouse IgG1 isotype. The mean tumor volumes ± SEM for the different treatment groups are shown. Arrows indicate dose administration timepoints for the first two weeks. At 63 days post-tumor regression (and 81 days after initiation of dosing), all tumor-free mice from the TroptimAb-SL-MMAE +anti-PD-1 combination cohort were rechallenged with EMT6.hTROP2^184K^ cells. Eight of 9 mice remained tumor-free at day 121 in the group that had previously received TroptimAb-SL-MMAE +anti-PD-1 combination.

### Summary of engineering and key TroptimAb-SL-MMAE properties

TroptimAb was engineered from sacituzumab with desired affinity, functional activity, and improved physicochemical profile using complementary computational engineering approaches and conventional laboratory yeast display techniques. The combination of changes identified in the lead variant from the two complementary campaigns resulted in an antibody with increased humanness, reduced hydrophobicity, and TROP2 targeting. The BSM variant’s eleven mutations derived from computational engineering collectively increased the percent identity to human germline, reduced hydrophobicity, and maintained the original affinity to human TROP2. The light chain Y53D mutation derived from yeast display reduced the affinity and avidity-tuned the antibody to lower binding to TROP2 high cells versus TROP2-low cells. Introduction of YTE mutations into the Fc region improved antibody exposure consistent with enhanced FcRn-mediated salvage from catabolism. The S375C mutation in the Fc region enabled site-specific conjugation with linker-MMAE using cysteine-maleimide chemistry. *In vivo* data obtained from xenograft efficacy studies run in mice combined with PK studies run in rhesus monkey supported the selection of the BSM rehumanized sequence, the avidity-tuned Y53D mutation, and FcRn-mediated recycling YTE mutations to be included in the final sequence of the antibody component of TroptimAb-SL-MMAE.

## Discussion

The TROP2 ADC landscape is dominated by Top1 inhibitor payloads with approved agents including sacituzumab govitecan, datopotamab deruxtecan, and sacituzumab tirumotecan alongside numerous clinical-stage candidates. In contrast, few TROP2 ADCs utilize alternative payloads highlighting opportunities for differentiated mechanisms and resistance-overcoming strategies (52). A TROP2-MMAE ADC was prospectively designed to help mitigate some of the known or hypothesized risks based on the known precedents from the ADC field. TroptimAb-MMAE was selected using *in vitro* and *in vivo* experimentation to be in an affinity range wherein it could effectively deliver MMAE to TROP2-high tumors while being less effective at binding and payload delivery to healthy TROP2-low cells.

Multiple modifications were incorporated into TroptimAb-SL-MMAE help minimize non-specific uptake due to hydrophobicity and slow down physiological antibody catabolism pathways (**Figure 11**). TroptimAb was derived from rehumanizing sacituzumab with an intended goal to reduce its abnormally high hydrophobicity profile; the eleven BSM mutations accomplished this goal. Amongst the potential mutations that could have been chosen to avidity-tune sacituzumab’s affinity, the Y53D mutation was chosen since it replaced a hydrophobic tyrosine with a charged aspartic acid leading to the least hydrophobic avidity-tuned antibody that was under consideration to convert to an ADC. The S375C site was chosen amongst other potential sites to allow efficient linker conjugation while also minimizing the hydrophobicity increase that would occur following MMAE conjugation. The S375C site is also sterically hidden within the Fc which provides an additional layer of shielding for the protease-cleavable linker from being cleaved by circulating extracellular proteases (e.g. differentiated neutrophil proteases). This feature biases the linker cleavage preferentially after internalization into the lysosomal compartment and loss of the antibody’s structural integrity within the lysosome. Lastly, the YTE mutation was incorporated into the Fc to promote FcRn-mediated salvage of internalized ADC leading to improved recycling back into circulation minimizing normal tissue catabolism and extended the ADC half-life. Endothelial cells and monocyte/macrophages have the highest FcRn message in normal organs in contrast to normal epithelial cells from those organs based on re-analysis of single cell data from Tabula Sapiens Consortium (53).; therefore, the largest change in ADC catabolism in normal tissue leading to increased systemic exposure by YTE incorporation may occur within these FcRn-high cell types. Altered ADC catabolism by YTE incorporation would also be expected in tumor tissue due to the presence of the same endothelial and monocyte/macrophage cells and their passive uptake of ADC. Antigen-dependent uptake of a TROP2 ADC would be less impacted by a tumor cell’s FcRn levels due to TROP2-mediated transport of the ADC to the lysosome. Incorporation of YTE into a TROP2 ADC did not impact the *in vitro* cytotoxicity potency in a panel of TROP2+ cell lines nor impact its *in vivo* efficacy after accounting for altered exposure in mice expressing mouse FcRn (data not shown). Overall, TroptimAb-SL-MMAE exhibited acceptable biophysical properties and ADC developability profile.

**Figure 11:**
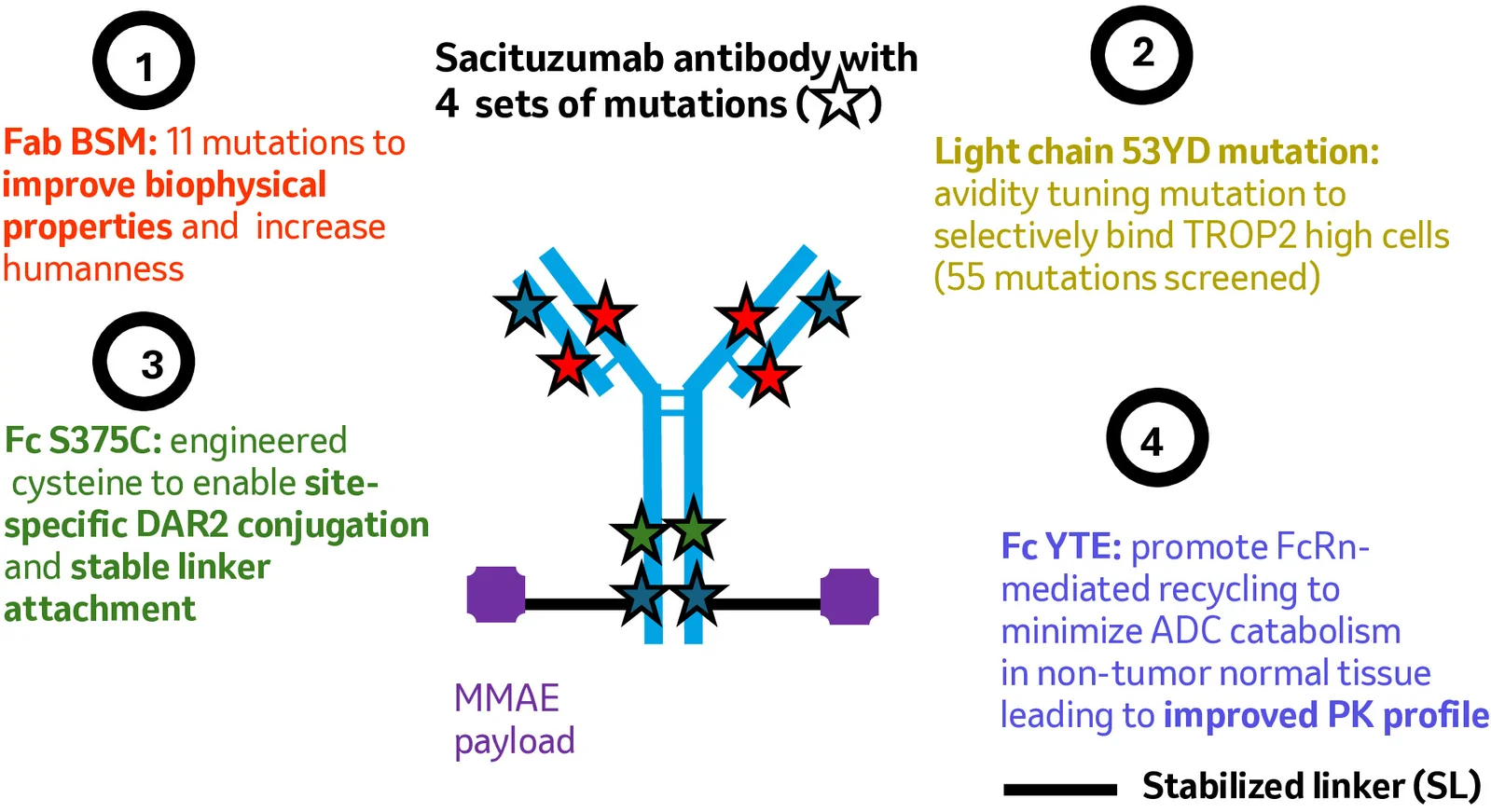
Summary of engineering design goals and key properties of TroptimAb-SL-MMAE. TroptimAb-SL-MMAE schematic highlighting sequence optimization features compared to sacituzumab. BSM=Best Single Mutations; DAR2=drug-antibody ratio of 2; MMAE=monomethyl auristatin E; PK=pharmacokinetics; S375C=serine to cysteine substitution at position 375; TROP2=trophoblast cell surface antigen 2; Y53D=tyrosine to aspartic acid substitution at position 53; YTE=methionine to tyrosine substitution at position 252, serine to threonine substitution at position 254, and threonine to glutamate substitution at position 256.

TroptimAb-SL-MMAE demonstrated single dose efficacy in TROP2-high xenograft models and showed a range of activity in a PDX panel that correlated with TROP2 membrane receptor density. Co-administration of TroptimAb-SL-MMAE with a PD-1 antagonist antibody showed combination benefit in a PD-1-resistant syngeneic tumor model. Importantly, mice that achieved complete response following co-administration developed immune memory that protected them from a subsequent tumor rechallenge. Companion studies were run in the same immune-competent model system using a different MMAE ADC (i.e. EMT6.hHER2 tumors treated with trastuzumab-MMAE (T-MMAE)) to explore the mechanistic basis for the enhanced response after MMAE ADC-based tumor cytotoxicity. T-MMAE treatment led to significant increase in immune cell infiltrate in the tumor. The increased tumor T-cell recruitment (CD4, CD8, and Treg) was similar to another ADC payload mechanism; however, T-MMAE selectively drove a significant myeloid cell recruitment including ClassI I+ macrophages. The macrophage influx correlated with increased expression of macrophage-associated genes such as *Adgre1* (F4/80), *Mrc1* (CD206), and *IL10RA* in the tumor. In addition, T-MMAE increased the tumor’s “T-cell inflamed IFN-g gene expression” signature (GEP) that is predictive of response to the PD-1 antagonist pembrolizumab (54). The tolerability and combination benefit of a MMAE ADC with a PD-1 antagonist antibody has already been shown in humans following the approval of PADCEV™ (Nectin4-MMAE) in combination with KEYTRUDA™ for the treatment of urothelial carcinoma (55).

There are multiple known or hypothesized resistance mechanisms that could minimize the efficacy of TroptimAb-SL-MMAE. Cancer cells may downregulate surface TROP2 expression, mutate TROP2 gene sequence to compromise Troptimab’s binding epitope on Trop2, or alter TROP2 internalization kinetics or intracellular destination away from the lysosome. Lysosomal protease levels or lysosomal pH levels could be altered to make the required linker cleavage step less efficient. Additionally, endogenous efflux pump levels (e.g. ABCB1/Pgp) could be increased to pump MMAE out of the cell or tubulin gene sequence mutated to compromise MMAE binding. Lastly, tumor cells may upregulate anti-apoptotic proteins or activate alternative survival signaling pathways to survive even when MMAE has caused mitotic arrest (56).

TROP2 is expressed in multiple cancer indications that have shown sensitivity to MMAE ADCs such as urothelial cancer (enfortumab vedotin), cervical cancer (tisotumab vedotin), gastric cancer (disitamab vedotin), wildtype EGFR NSCLC (telisotuzumab vedotin), and head and neck squamous cell carcinoma (HNSCC) (becotatug vedotin). In addition, combining ADCs that carry different payloads mechanisms such as topo1 inhibitors, anthracyclines, anti-metabolites, alkylating agents, or combining with untargeted chemotherapy may produce increased efficacy by targeting different phases of the cell cycle (57). The first ADC combination study was the phase I Double Antibody Drug Conjugate (DAD) trial to assess the safety and combination maximum tolerated doses of sacituzumab govitecan and enfortumab vedotin in patients with treatment-resistant metastatic urothelial cancer leading to a 70% overall response rate (58). Datopotamab deruxtecan and sacituzumab tirumotecan are being evaluated in combination with the doublet of PD-1 antagonist and untargeted carboplatin/cisplatin in NSCLC; sacituzumab govitecan is being evaluated with the untargeted anti-metabolite capecitabine.

Clinical studies will determine the range of clinical efficacy in TROP2+ indications that a TROP2-targeted MMAE can achieve at tolerated doses using the avidity tuning strategies employed here to mitigate the anticipated on-target and off-target toxicities. Conceptually, tumor indications with higher TROP2 expression levels (e.g. cervical, bladder, HNSCC; **Supplementary Figure 1**) would be preferentially targeted by TroptimAb-SL-MMAE avidity tuning strategy as was seen with PDX tumors expressing higher versus low TROP2 levels. MMAE was chosen as the payload based on its known bystander killing property that mitigates intra-tumoral TROP2 expression heterogeneity within a lesion. Early clinical studies would include retrospective analysis of TROP2 expression in patient biopsies (both expression magnitude and expression heterogeneity) as a potential prospective response biomarker; these information would inform on a prospective IHC expression threshold needed for a patient to have obtain maximal therapeutic benefit. Post-dose biopsy collection would allow assessment of pharmacodynamic endpoints of efficient MMAE delivery into tumor cells (e.g. pHH3 and CC3); selection of biopsy collection timepoints after dosing would be critical due to the temporal induction and resolution of these pharmacodynamic endpoints. .

## Data Availability

The datasets presented in this study can be found in online repositories. The names of the repository/repositories and accession number(s) can be found in the article/**Supplementary Material**.

## Supplementary material

Supplementary Figure 1 Gene and IHC expression demonstrating that TROP2 is widely expressed in human tumors and in normal tissue from humans and rhesus non-human primates: **(A)** TROP2 gene expression in a panel of human tumors, **(B)** TROP2 gene expression in a panel of healthy normal tissues, **(C)** Membrane TROP2 protein expression in different tumor indications as assessed by H-score metric, **(D)** Representative IHC pictures of TROP2 protein expression in various healthy human normal tissues. The epithelial population was TROP2+ in esophagus (all squamous cells), skin (all keratinocytes), bladder (all transitional cells), lung (most bronchiolar and alveolar epithelial cells), kidney (most epithelial cells of a subset of proximal convoluted tubules), pancreas (most acinar and islet cells), stomach (small subset of foveolar glandular cells), and cornea (all corneal epithelial cells). **(E)** Representative IHC pictures of TROP2 protein expression in various healthy rhesus non-human primate normal tissues.

Supplementary Figure 2 Sacituzumab NHP PK profile and PK parameters **(A)** Individual plasma concentration-time profiles of sacituzumab in rhesus monkeys (n = 2) at 0.1 mg/kg (red), 1 mg/kg (green), or 3 mg/kg (blue). Symbols (measured data); Lines (fitted data using a two compartmental PK/TMDD model). LLOQ: lower limit of quantitation. **(B)** Two compartmental PK/TMDD model schematics and fitted PK parameters using Pheonix (Certara Inc.)

Supplementary Figure 3 Dose-dependent temporal changes in pharmacodynamic biomarkers pHH3 and CC3 following TroptimAb-SL-MMAE treatment of BxPC3 tumors. BALB/c nude mice implanted with BxPC-3 cells subQ received IV administration of a single dose at Day 0 of PBS or TroptimAb-SL-MMAE at 1.25, 2.5, 5, or 10 mg/kg. Tumors were harvested at the indicated timepoints, fixed in 10% formalin overnight, and embedded in paraffin. FFPE sections were stained for pHH3 or CC3 biomarkers. The % of tumor cells with pHH3+ nuclei was quantified using HALO image analysis software; CC3 staining was manually assessed by a pathologist using a qualitative scoring system (0 = negative, 1 = minimal, 2 = low, 3 = moderate, 4 = high, 5 = very high)

Supplementary Figure 4 Sacituzumab govitecan and datopotamab deruxtecan anti-tumor efficacy in PDX models. **(A)** Summary of TROP2 gene expression and key efflux pump gene expression of 11 PDX models tested. PDX expression data were generated by and obtained from Crown Bioscience Inc. **(B)** PDX models established in immunodeficient BALB/c nude or NOD/SCID mice and treated with IV administration of 2 doses of PBS at Days 0 and 21, 10 mpk datopotamab deruxtecan at Days 0 and 21, or 20 mpk sacituzumab govitecan twice weekly for 4 weeks (n=5 mice per treatment group). Arrows denote treatment at Days 0 and 21. PDX TROP2 H-score (0-300): BR1282 TNBC (300), BL5003 bladder (300), LU0861 NSCLC (300), LU0387 NSCLC (300), LU2049 NSCLC (300), LU11694 NSCLC (300), BL9214 bladder (228), BR1458 TNBC (0).

Supplementary Table 1 Summary of sacituzumab and its avidity tuned variants’ properties: differential cell binding to cells lines with different TROP2 levels, affinity to human TROP2 by SPR, purity by CE-SDS, aggregation measured by UP-SEC, thermostability (Tonset, Tm1, Tagg) measured by nano-DSF, and hydrophobicity measured by HIC using the Propac method/column.

Supplementary Table 2 Affinity of Troptimab-SL-MMAE to various species of TROP2 measured by SPR. Binding to canine TROP2 was assayed with immobilized TROP2, The other species were tested with an anti-HuFc capture.

Supplementary Table 3 TroptimAb-SL-MMAE average pharmacokinetic parameters following intravenous administration to female nude mice bearing BxPC3 xenograft tumor.

## Glossary

ADC: Antibody-drug conjugate
AEX: Anion exchange chromatography
BSM: best single mutant
cAb: conjugated antibody
CDR: Complementary-Determining Region
CE-SDS: capillary electrophoresis-sodium dodecyl sulfate
CHO: Chinese Hamster Ovary
cIEF: Capillary isoelectric focusing
CMC: Chemistry, Manufacturing, and Controls
Cp: Centipoise
DLS: Dynamic light scattering
DSF: Differential scanning fluorimetry
Fc: Fragment crystallizable region of an antibody
FcγR: Fc gamma receptor
FcRn: Neonatal Fc receptor
HER2: Human Epidermal growth factor Receptor 2
HIC: Hydrophobic interaction chromatography
His: Histidine
HMW: High molecular weight
HNSCC: Head and neck squamous cell carcinoma
HR: hormone receptor
IEX: Ion-exchange chromatography
IgG1: human immunoglobulin of IgG1 subclass
IHC: immunohistochemistry
K_D_: Binding affinity constant
LC-MS: Liquid chromatography-mass spectrometry
M: Molar
mAb: Monoclonal antibody
MMAE: Monomethyl auristatin E
Nano-DSF: nano Differential Scanning Fluorimetry
NHP: Non-Human Primate
NSCLC: non-small cell lung cancer
PABC: para-aminobenzyl carbamate
PDX: patient-derived xenograft
pI: Isoelectric point
PTM: Post translational modifications
RP-HPLC: Reverse Phase High-Performance Liquid Chromatography
RT: Retention time
SPR: Surface plasmon resonance
SL-MMAE: Stabilized Linker- MMAE payload
TAA: Tumor-associated antigen
TAb: Total antibody
TACSTD2: Tumor-associated calcium signal transducer 2
Tagg: Temperature of aggregation onset
Tm: Thermal unfolding transition midpoint
TMDD: Target-mediated drug disposition
Tonset: Temperature of melting onset
TROP2: Trophoblast cell surface antigen 2
TroptimAb sacituzumab containing BSM: Y53D, YTE, S375 mutations
TroptimAb-SL-MMAE: ADC made with Troptimab sequence and stabilizedMMAE linker/payload
UP-SEC: Ultra pressure-size exclusion chromatography
VH: Variable domain of heavy chain
VL: Variable domain of light chain
